# Pooled Sequencing of Candidate Genes Implicates Rare Variants in the Development of Asthma Following Severe RSV Bronchiolitis in Infancy

**DOI:** 10.1371/journal.pone.0142649

**Published:** 2015-11-20

**Authors:** Dara G. Torgerson, Tusar Giri, Todd E. Druley, Jie Zheng, Scott Huntsman, Max A. Seibold, Andrew L. Young, Toni Schweiger, Huiqing Yin-Declue, Geneline D. Sajol, Kenneth B Schechtman, Ryan D. Hernandez, Adrienne G. Randolph, Leonard B. Bacharier, Mario Castro

**Affiliations:** 1 Department of Medicine, University of California San Francisco, San Francisco, California, United States of America; 2 Department of Internal Medicine, Washington University School of Medicine, St. Louis, Missouri, United States of America; 3 Center for Genome Sciences and Systems Biology, Washington University School of Medicine, St. Louis, Missouri, United States of America; 4 Department of Biostatistics, Washington University School of Medicine, St. Louis, Missouri, United States of America; 5 Integrated Center for Genes, Environment and Health, National Jewish Health, Denver, Colorado, United States of America; 6 Department of Bioengineering and Therapeutic Sciences, Institute of Human Genetics, and California Institute of Quantitative Biosciences (QB3), University of California San Francisco, San Francisco, California, United States of America; 7 Department of Anesthesiology, Boston Children’s Hospital, Boston, Massachusetts, United States of America; 8 Department of Pediatrics, Washington University School of Medicine and St. Louis Children’s Hospital, St. Louis, Missouri, United States of America; Imperial College London, UNITED KINGDOM

## Abstract

Severe infection with respiratory syncytial virus (RSV) during infancy is strongly associated with the development of asthma. To identify genetic variation that contributes to asthma following severe RSV bronchiolitis during infancy, we sequenced the coding exons of 131 asthma candidate genes in 182 European and African American children with severe RSV bronchiolitis in infancy using anonymous pools for variant discovery, and then directly genotyped a set of 190 nonsynonymous variants. Association testing was performed for physician-diagnosed asthma before the 7^th^ birthday (asthma) using genotypes from 6,500 individuals from the Exome Sequencing Project (ESP) as controls to gain statistical power. In addition, among patients with severe RSV bronchiolitis during infancy, we examined genetic associations with asthma, active asthma, persistent wheeze, and bronchial hyperreactivity (methacholine PC_20_) at age 6 years. We identified four rare nonsynonymous variants that were significantly associated with asthma following severe RSV bronchiolitis, including single variants in *ADRB2*, *FLG* and *NCAM1* in European Americans (p = 4.6x10^-4^, 1.9x10^-13^ and 5.0x10^-5^, respectively), and *NOS1* in African Americans (p = 2.3x10^-11^). One of the variants was a highly functional nonsynonymous variant in *ADRB2* (rs1800888), which was also nominally associated with asthma (p = 0.027) and active asthma (p = 0.013) among European Americans with severe RSV bronchiolitis without including the ESP. Our results suggest that rare nonsynonymous variants contribute to the development of asthma following severe RSV bronchiolitis in infancy, notably in *ADRB2*. Additional studies are required to explore the role of rare variants in the etiology of asthma and asthma-related traits following severe RSV bronchiolitis.

## Introduction

Asthma is a complex disease caused by both genetic and environmental factors, and the interactions between them.[1] Among the many environmental risk factors for asthma are viral respiratory infections, which are important, common triggers of asthma exacerbations in children.[2,3] Bronchiolitis due to respiratory syncytial virus (RSV) is a leading cause of hospitalization among infants and children before the age of two.[4] Multiple studies have reported that infants hospitalized for severe RSV bronchiolitis are at significantly increased risk for wheezing illness and asthma later in childhood.[5–8] However, not all children exposed to RSV experience subsequent recurrent wheezing illness, and not all children who experience subsequent episodes of wheezing illness are eventually diagnosed with asthma, suggesting that genetic factors may also play a role in this phenomenon.

More than 100 genes have been associated with asthma and asthma-related traits, however there is marked variability in replication attempts in independent studies.[9] This may in part be due to low statistical power from small samples, inadequate control for multiple comparisons, heterogeneity in environmental exposures and outcome measurement, and differences in study design. Genome-wide association (GWA) studies have focused on the search for common genetic risk variants (allele frequencies > 5%) that influence complex diseases.[10] Nevertheless the risk alleles identified through these studies have explained only a small proportion of the heritability of this complex disease.[11]

The evolution of the common disease/rare variant hypothesis suggests that the majority of the missing heritability in common and complex phenotypes is instead due to rare or private DNA variants.[10] Previous studies have implicated rare variants in asthma and asthma related traits for a number of relevant genes,[12–15] suggesting that rare variants may indeed play an important role in asthma susceptibility; however, this has not been studied in the context of severe RSV bronchiolitis in infancy. In the current study, we performed pooled anonymous sequencing of coding exons from 131 asthma candidate genes in 182 individuals with severe RSV bronchiolitis in infancy from the RSV Bronchiolitis in Early Life (RBEL) study [16] for variant discovery. We then genotyped a set of 190 nonsynonymous variants, and tested for allelic associations with physician-diagnosed asthma before the 7^th^ birthday (asthma) following severe RSV bronchiolitis in infancy using the ESP as controls to gain statistical power. Lastly, among patients with severe RSV bronchiolitis during infancy, we examined genetic associations with asthma, active asthma, persistent wheeze, and bronchial hyperreactivity (methacholine PC_20_) at age 6 years.

## Materials and Methods

### Study populations

#### RBEL

In the RSV Bronchiolitis in Early Life (RBEL) prospective cohort study, 206 infants (12 months of age or less) were enrolled from 1998 to 2001, of which 182 had DNA samples available and were included in the current study (Table 1). Complete selection criteria for the study population and characteristics of the cohort at study entry are described in detail elsewhere.[17] Included infants were required to have bronchiolitis severe enough to require emergency department care or hospitalization, a positive nasopharyngeal swab result confirming infection with RSV, and physician-documented wheezing during the acute illness. Exclusion criteria were a history of previous wheezing or a diagnosis of asthma, congenital abnormalities of the heart and lung, cystic fibrosis diagnosed in the patient or immediate family, regular use of anti–gastroesophageal reflux medication, bronchodilators, or anti-inflammatory medications.

**Table 1:** Summary of clinical characteristics of participants in the RBEL study.

	Physician-Diagnosed Asthma (N = 96)	No Asthma (N = 96)
	**Demographics**	
**Age at enrollment, days**	122 ± 107	152 ± 107
**Male (%)**	55.2 (53)	62.8 (54)
**Caucasian (%)**	47.9 (46)	61.6 (53)
	**Hospitalization Data**	
**Length of stay in hospital (days)**	2.6 ± 2.5	2.4 ± 2.7
**Lowest SaO**_**2**_ **in hospital (%)**	92 ± 6	91 ± 8
	**Family History**	
**Maternal history of asthma (%)**	26.0 (25)	6.98 (6)
**Maternal history of eczema (%)**	5.21 (5)	8.14 (7)
**Maternal history of allergic rhinitis (%)**	26.0 (25)	14.0 (12)
	**Clinical History and Other Exposures**	
**Smoking in home during 1** ^**st**^ **year of life (%)**	64.2 (61)	72.3 (60)
**Dog in home (%)**	42.7 (41)	40.2 (33)
**At least one positive skin test to aeroallergen at 3 years of age (%)**	39.7 (31)	22.2 (14)
	**Lab Tests at Entry**	
**IgE (IU/mL)**	25.7 ± 53.7	20.6 ± 33.2
**Blood Eosinophils (%)**	1.64 ± 2.39	2.08 ± 2.85
	**Lung Function at Age 6**	
**FEV**_**1**_ **%predicted, pre-BD**	99 ± 15	103 ± 18
**FEV**_**1**_ **/FVC, pre-BD**	0.91 ± 0.10	0.90 ± 0.11
**% change in FEV**_**1**_ **post-BD**	4.83 ± 13.2	4.08 ± 10.3
**Methacholine PC**_**20**_ **(mg/mL)**	0.56 ± 0.67	1.08 ± 1.26

Abbreviations used: SaO_2_ = lowest oxygen saturation recorded during index hospitalization; IgE = immunoglobulin E; FEV_1_ = forced expiratory volume in one second; BD = bronchodilator; PC_20_ = provocative concentration of methacholine that causes a 20% decline in FEV_1._

A child was classified as having **physician-diagnosed asthma** (henceforth, “**asthma**”) if the parent/guardian answered “Yes” to “Has your child ever been diagnosed with asthma by a physician?” at any time before the child’s 7^th^ birthday. A persistent “No” response defined children without physician diagnosed asthma. We defined **active asthma** as physician-diagnosed asthma at any time along with parent-reported wheezing during the last year of follow-up between the child’s 3^rd^ and 7^th^ birthdays. We defined **persistent wheezing** as having at least one wheezing episode in first three years of life and at least one wheezing episode between the child’s 3^rd^ and 7^th^ birthdays. Methacholine bronchoprovocation was performed at age 6 as previously reported.[17] We defined **bronchial hyperreactivity** as the provocation concentration of inhaled methacholine that caused a 20% drop in baseline forced expiratory volume in one second (methacholine PC_20_).

This study was approved by the Washington University School of Medicine Institutional Review Board (RBEL), and the Boston Children’s Hospital Institutional Review Board (BRASS). Written informed consent was obtained from parents or guardians.

#### BRASS

A detailed description of the Boston RSV bronchiolitis cohort (BRASS) is available elsewhere.[18] Briefly, 207 otherwise healthy children hospitalized for RSV bronchiolitis at Boston Children’s Hospital were identified from the hospital database of positive RSV laboratory test results (rapid test and/or culture) and were recruited retrospectively. Self-reported non-Hispanic White individuals from the BRASS study were included for replication of the findings from the RBEL cohort if they were hospitalized before 1 year of age, to be consistent with the RBEL enrollment criteria.

### DNA sequencing

A total of 99 European Americans (EA) and 83 African Americans (AA) from the RBEL study were sequenced in four anonymous pools for variant discovery. These pools were “anonymous” in that DNA from a single individual was not barcoded prior to pooling DNA for sequencing, and thus the resulting variants could not be assigned to a single individual (but rather to a pool of individuals). Genomic DNA was extracted from whole blood using Wizard genomic DNA purification kit (Promega) and individually quantified by NanoDrop 2000 spectrophotometer (Thermo Scientific). Insufficient DNA yield samples (n = 65) were amplified using REPLI-g Mini Kit (Qiagen Inc) and subsequently purified using QIAamp DNA blood mini kit (Qiagen Inc). 200 ng DNA from each individual was combined into four separate anonymous pools, with separate pools for European and African Americans, and for asthma cases and asthma controls.

A total of 131 candidate genes were selected from published studies that demonstrated a prior, positive association with asthma (S1 Table). Coding exons of 131 genes were individually PCR amplified using custom primers designed using Primer3 (frodo.wi.mit.edu/), and using *in silico* PCR on the UCSC Genome Browser[19,20] to confirm the specificity of each primer pair (S2 Table). PCR amplification using 40 ng of pooled DNA per reaction and preparation of the PCR products for sequencing on the Illumina Genome Analyzer I or II was performed as previously described.[21] Briefly, amplicons were purified from primers and residual nucleotides by Qiaquick column separation (Qiagen), combined into mixtures containing an equivalent number of molecules of each amplicon (1x10^11^), and concatenated overnight at 22°C with T4 DNA ligase and T4 polynucleotide kinase (New England Biolabs) in the presence of 15% (w/v) polyethylene glycol, MW8000 (Sigma-Aldrich).

Positive and negative control DNA amplicons were included in ligations to monitor base calling accuracy.[22] After 10-fold dilution in buffer PB (Qiagen), random fragmentation by sonication (Diagenode Bioruptor XL), and purification on Qiaquick columns (Qiagen Inc), DNA sequencing libraries for each pool were prepared according to Illumina protocols. Libraries were sequenced on the Illumina Genome Analyzer IIx platform in Washington University’s Genome Technology Analysis Center.

### Sequence Analysis and Variant Calling

Sequence analysis and variant calls was performed using SPLINTER.[22] A model of sequencing error was determined by the analysis of a non-variant 1,934 bp amplified sequence from the pGem-T Easy backbone, which was incorporated into each library. Sensitivity was determined by the ability to identify variants in positive controls. These controls dictated p-values for variant calling from each individual set of raw sequence data, and were then used for variant calling within the remaining reads that uniquely aligned to targeted sequences. Sequencing output was aligned to the human reference genome (hg19) allowing for 2 mismatches or fewer for each read. Any read with more than 2 mismatches or that aligned to multiple locations in hg19 was discarded.

### Genotyping and Quality Control

We performed custom genotyping of 384 variants using an Illumina GoldenGate array for all individuals within each of the four anonymous pools. A total of 710 unique nonsynonymous variants were identified through pooled sequencing, and scored as to their designability for the GoldenGate array (https://icom.illumina.com/Custom/UploadOpaPrelim/). A total of 384 variants in coding exons were chosen for custom array design (designability rank 1; n = 309, and designability rank 0.5; n = 75), which included 190 nonsynonymous variants. Genotyping was performed concurrently on all 182 individuals from the RBEL study (the discovery cohort), and 177 individuals from the BRASS study (the replication cohort).

Genotyping on the custom GoldenGate array (Illumina Inc, San Diego) was performed according to the manufacturer’s Instructions (ref. GoldenGate Genotyping Assay Guide 15004065 B). An iScan System was used for chip scan and image acquisition. Genotypes were called using Genome Studio v.2009.1 software (Illumina Inc), and quality control was performed using PLINK.[23] Subjects were filtered based on call rate < 90% (n = 4 subjects from RBEL, n = 12 subjects from BRASS), and variants were filtered based on call rate < 95% and/or Hardy-Weinberg p-value < 0.05 within each self-reported ethnicity/study. Carriers of four associated rare variants underwent additional validation using Sanger sequencing.

### Association Testing

Association testing at individual variants genotyped on the GoldenGate array was performed using logistic or linear regression within each ethnicity in PLINK.[23] We tested for an association with asthma following severe RSV bronchiolitis in infancy vs. population controls from the exome sequencing project [ESP]. Variants identified using the ESP as controls were further examined for an association with asthma, active asthma, persistent wheeze, and bronchial hyperreactivity at age 6 (methacholine PC_20_, log transformed) among individuals with severe RSV bronchiolitis in infancy (excluding the ESP). Covariates examined included age at severe RSV bronchiolitis, sex, daycare attendance, maternal smoking during pregnancy and at time of enrollment, smoke exposure from other family members in home, and asthma status for bronchial hyperreactivity.

Genotype counts from the ESP were downloaded from the NHLBI Exome Sequencing Project Exome Variant Server (ESP6500SI-V2)[24], converted into PLINK format using custom perl scripts, and merged with genotypes from the RBEL study. A total of 17 variants showing an association at p<0.01 with asthma following severe RSV were subject to additional QC in the RBEL study by visual inspection of cluster plots for GoldenGate genotypes, and comparisons of allele frequencies from GoldenGate genotyping vs. those estimated from pooled anonymous sequencing. Any variants that showed questionable distinction between genotype clusters, or were discordant in frequency from pooled sequencing vs. direct genotyping whereby the frequency from pooled sequencing was more similar to the ESP were removed from further consideration (N = 12). Replication for asthma following severe RSV bronchiolitis vs. no asthma was similarly performed using logistic regression on genotypes obtained from the same custom GoldenGate array in the BRASS study.

## Results

**Fig 1:**
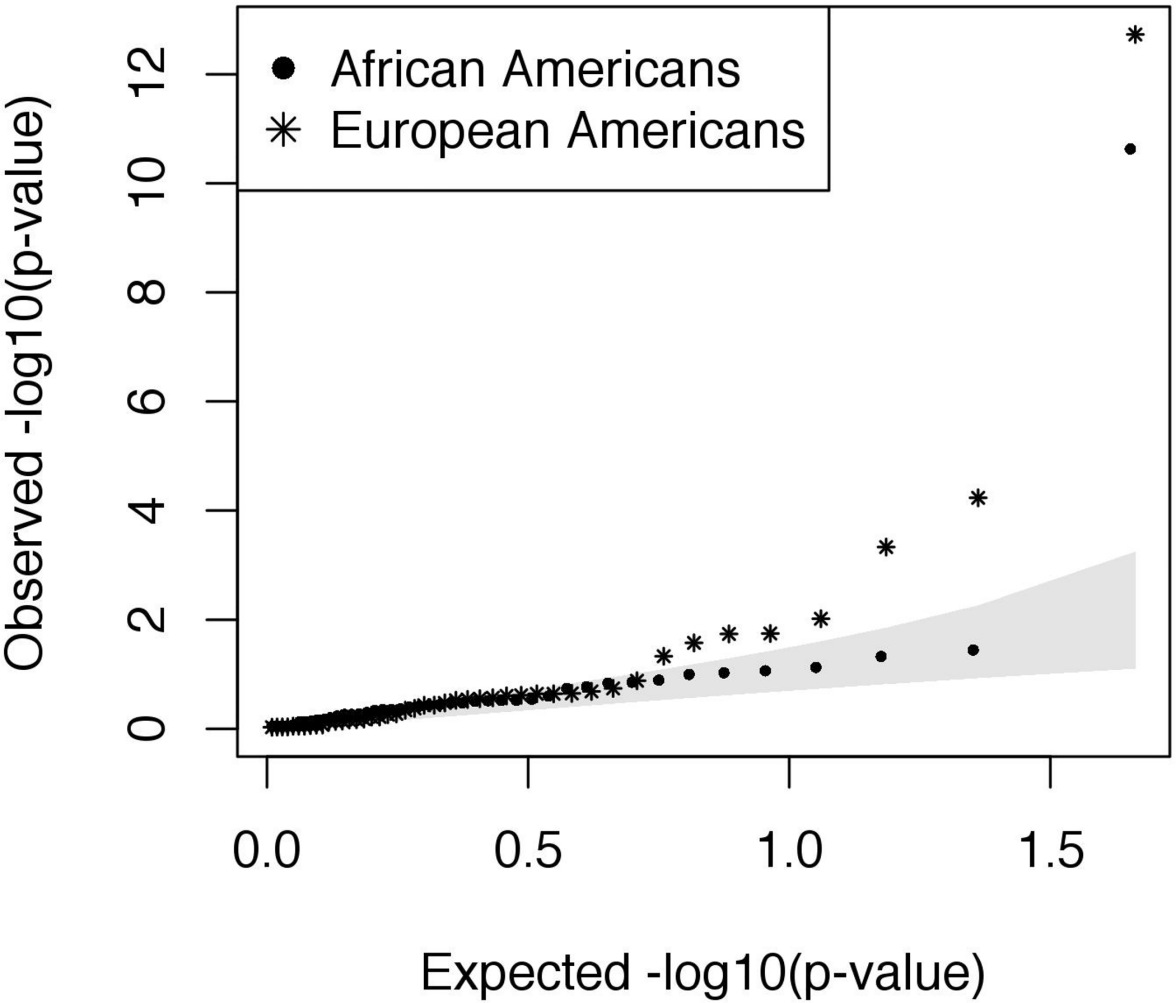
Iceberg plot showing how the majority of coding variants identified per gene are at minor allele frequencies (MAF) at or below 10% in both European American (EA, N = 39) and African American (AA, N = 49) asthma cases and controls following severe RSV bronchiolitis in infancy. A total of 131 asthma genes were sequenced; each vertical bar represents a gene with at least one coding variant detected in the sample.

We obtained a total of 695,366 base pairs (bp) of targeted sequencing at an average depth of 52–353x coverage/allele at variable sites in 182 individuals in the RBEL study (Table 2). We observed differences in sequencing coverage across pools, which could have lead to differences in the number of variant calls between pools (Table 2). Therefore, we calculated a p-value threshold for variant calls using internal positive and negative controls within each pool rather than across pools to best account for this. Specifically, we normalized the p-value threshold within each pool by sequencing coverage to maximize sensitivity and specificity. Using the SPLINTER algorithm, we identified a total of 5,496 single nucleotide variants (SNVs), including 710 nonsynonymous, 441 synonymous, and 4,372 non-coding variants. As expected, the majority of variants were at low frequency or rare (Fig 1, Table 2), and many of the rare variants were private to asthma cases or controls within each population (Table 3).

**Table 2:** Overview of total and rare variants called in anonymous pooled sequencing of coding exons for 131 candidate genes using the SPLINTER algorithm. EA = European American, AA = African American, MAF = minor allele frequency.

Pool	Population	N	Aligned Reads (million)	Total Variants	Total Rare Variants (MAF<5%)	Mean Coverage/Allele at Variable Sites
1	EA Cases	39	32.1	2,673	1,573	52
2	AA Cases	48	19.4	3,432	1,567	72
3	EA Controls	60	21.8	2,258	883	87
4	AA Controls	34	20.4	1,123	236	353
	TOTAL	181	93.7	5,496	3,433*	

*MAF<5% in at least one pool.

**Table 3:** Counts of rare and private rare variants in asthma cases vs. controls in anonymous pooled sequencing of coding exons of 131 candidate genes. Private variants are those private to either cases or controls within an ethnicity. NS = nonsynonymous, Syn = synonymous, NC = non-coding.

		Rare Variants			Private Rare Variants		
	Pool	NS	Syn	NC	NS	Syn	NC
African Americans:	Cases (N = 48)	258	129	1180	230	99	1049
	Controls (N = 34)	32	30	174	10	16	79
European Americans:	Cases (N = 39)	213	72	1288	180	43	1081
	Controls (N = 60)	95	71	717	71	51	471

A total of 384 rare and common variants, including 190 nonsynonymous variants were genotyped directly in the same samples included in the anonymous pools on a custom GoldenGate array. Of these, 264 variants passed stringent quality control filters. Of the 120 variants failing QC, 76 had call rates below 95%, 61 had Hardy-Weinberg p-values < 0.05 in the African American samples, and 38 had Hardy-Weinberg p-values < 0.05 in the European American samples. Not all variants identified through sequencing were identified as being polymorphic on the GoldenGate array, which may be due to the variants being absent (false positive calls) or difficulties in clustering carriers of rare variants (false negative calls). However, variants were confirmed, and carriers of minor/rare alleles were identified at 78 variants in the African American samples, and 49 variants in the European American samples. Individuals from the replication study (BRASS, European Americans) were genotyped using the same custom array using whole genome amplified DNA; 275 variants passed QC (87 had call rates below 95%, 72 had Hardy-Weinberg p-values < 0.05, 247 of which also passed QC in RBEL); a total of 76 variants were found to be polymorphic in BRASS. Across both studies combined (RBEL and BRASS), a total of 92 variants were brought forward for association testing.

**Fig 2:**
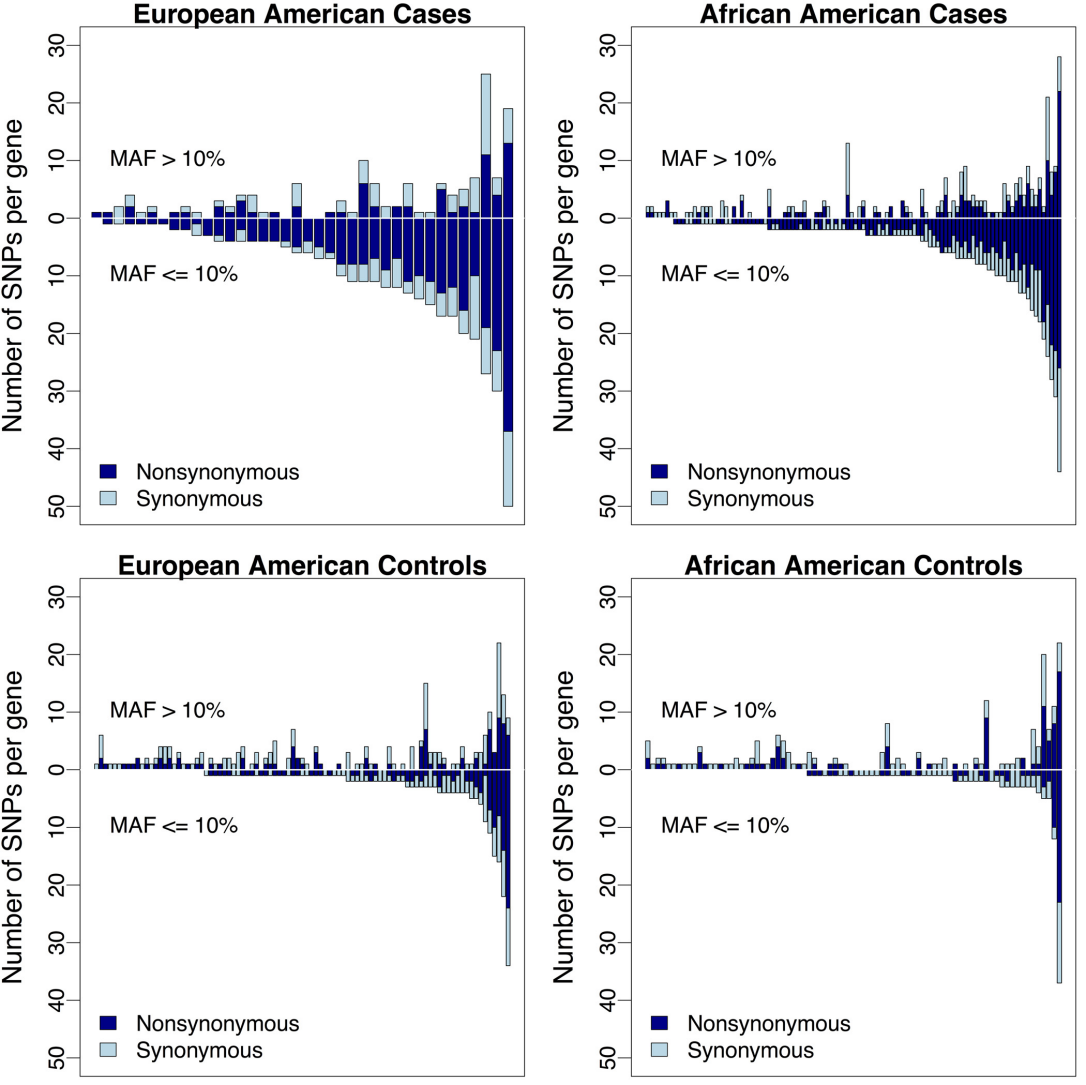
QQplot showing the results of association testing at nonsynonymous variants for physician-diagnosed asthma following severe RSV bronchiolitis in infancy. Genotypes from the exome sequencing project (ESP) were used as controls. The shaded area represents the 95% confidence interval.

To have sufficient statistical power, we included 6,503 individuals from the Exome Sequencing Project (ESP6500) as controls, including 2,203 African Americans and 4,300 European Americans. Following additional QC, allele frequencies in asthma cases from the RBEL study were in general consistent with frequencies in the ESP (Fig 2). However, we identified four nonsynonymous variants that were significantly associated with increased risk of asthma following severe RSV bronchiolitis in infancy, including one in African Americans in *NOS1* (Bonferroni threshold: α_78_ = 6.4x10^-4^) and three in European Americans in *FLG*, *ADRB2*, and *NCAM1* (α_49_ = 1.0x10^-3^) (Table 4). All of the variants were low frequency (MAF<5%) or rare (MAF<1%) in the ESP. Rs1800888 in *ADRB2* was also nominally associated with asthma (p = 0.027) and active asthma (p = 0.013) among European Americans with severe RSV bronchiolitis in infancy, without including the ESP as controls (Table 5). Rs35576001in *NCAM1* was associated with asthma in European Americans including the ESP as controls (rs35576001, p = 5.0x10^-5^, Table 4), however, the same variant was protective for persistent wheeze among African Americans with severe RSV bronchiolitis in infancy (p = 0.021, Table 5). For all but rs1800888, allelic associations were driven by a single carrier of the minor allele in asthma cases with severe RSV bronchiolitis in infancy, with no individual carrying >1 associated variant. Further validation of these variants through Sanger sequencing in asthma cases confirmed the presence of the minor allele in 6 carriers of rs1800888 in *ADRB2* (European Americans), one carrier of rs35576001 in *NCAM1* (a European American), and one carrier of rs76090928 in *NOS1* (an African American).

**Table 4:** Results of allelic association testing for physician-diagnosed asthma following severe RSV bronchiolitis in infancy including genotypes from the Exome Sequencing Project (ESP) as controls.

				RBEL European Americans:			RBEL African Americans:		
Position (hg19)	rsID	Gene	Alleles (A1/A2)	Freq A1 asthma cases	Freq A1 ESP	P-value ESP	Freq A1 asthma cases	Freq A1 ESP	P-value ESP
chr1:152283023	NA	*FLG*	T/A	0.013	0.0001	1.9x10^-13^	0	0	NA
chr5:148206885	rs1800888	*ADRB2*	T/A	0.064	0.015	4.6x10^-4^	0	0.0032	0.58
chr11:113076804	rs35576001	*NCAM1*	A/G	0.013	0.0006	5x10^-5^	0.021	0.027	0.74
chr12:117768154	rs76090928	*NOS1*	A/G	0.013	0.004	0.23	0.011	0	2.3x10^-11^

**Table 5:** Results of further association testing of candidate variants within individuals with severe RSV bronchiolitis in infancy, without including genotypes from the ESP. Phenotypes include physician diagnosed asthma, active asthma, persistent wheeze, and methacholine PC_20_ at age 6 years. P-values < 0.05 are in bold italics, Freq = allele frequency, OR = odds ratio, SE = standard error.

***Physician Diagnosed Asthma*:**									
				**European Americans: 39 cases, 60 controls**			**African Americans: 49 cases, 34 controls**		
**Position (hg19)**	**rsID**	**Gene**	**Alleles (A1/A2)**	**Freq A1 cases**	**Freq A1 controls**	**p-value (OR)**	**Freq A1 cases**	**Freq A1 controls**	**p-value (OR)**
chr1:152283023	NA	*FLG*	T/A	0.013	0	0.22 (NA)	0	0	NA
chr5:148206885	rs1800888	*ADRB2*	T/A	0.064	0.0085	***0*.*027*** (8.0)	0	0	NA
chr11:113076804	rs35576001	*NCAM1*	A/G	0.013	0	0.22 (NA)	0.021	0.015	0.78 (1.4)
chr12:117768154	rs76090928	*NOS1*	A/G	0.013	0	0.22 (NA)	0.011	0	0.40 (NA)
***Active Asthma*:**									
				**European Americans: 16 cases, 49 controls**			**African Americans: 26 cases, 25 controls**		
**Position (hg19)**	**rsID**	**Gene**	**Alleles (A1/A2)**	**Freq A1 cases**	**Freq A1 controls**	**p-value (OR)**	**Freq A1 cases**	**Freq A1 controls**	**p-value (OR)**
chr1:152283023	NA	*FLG*	T/A	0	0	NA	0	0	NA
chr5:148206885	rs1800888	*ADRB2*	T/A	0.063	0	***0*.*013*** (NA)	0	0	NA
chr11:113076804	rs35576001	*NCAM1*	A/G	0.031	0	0.079 (NA)	0.019	0.02	0.98 (0.96)
chr12:117768154	rs76090928	*NOS1*	A/G	0	0	NA	0.02	0	0.31 (NA)

***Persistent Wheeze*:**									
				**European Americans: 57 cases, 36 controls**			**African Americans: 50 cases, 19 controls**		
**Position (hg19)**	**rsID**	**Gene**	**Alleles (A1/A2)**	**Freq A1 cases**	**Freq A1 controls**	**p-value (OR)**	**Freq A1 cases**	**Freq A1 controls**	**p-value (OR)**
chr1:152283023	NA	*FLG*	T/A	0.0088	0	0.43 (NA)	0	0	NA
chr5:148206885	rs1800888	*ADRB2*	T/A	0.035	0.014	0.38 (2.6)	0	0	NA
chr11:113076804	rs35576001	*NCAM1*	A/G	0.0088	0	0.43	0	0.053	***0*.*021*** (0)
chr12:117768154	rs76090928	*NOS1*	A/G	0.0088	0	0.43	0.010	0	0.53 (NA)

***Bronchial Hyperreactivity (logPC***_***20***_ ***)*:**									
				**European Americans: N = 67**			**African Americans: N = 51**		
**Position (hg19)**	**rsID**	**Gene**	**Allele (A1)**	**Beta**	**SE**	**p-value**	**Beta**	**SE**	**p-value**
chr1:152283023	NA	*FLG*	T	1.0	1.3	0.43	NA	NA	NA
chr5:148206885	rs1800888	*ADRB2*	T	-0.37	0.66	0.58	NA	NA	NA
chr11:113076804	rs35576001	*NCAM1*	A	-0.18	1.3	0.89	-0.45	1.1	0.69
chr12:117768154	rs76090928	*NOS1*	A	NA	NA	NA	0.21	1.1	0.86

Replication of four variants associated with physician-diagnosed asthma was attempted in an independent study (BRASS, N = 63 asthma cases and 102 controls of European American ethnicity, Table 6). Genotypes from the ESP were not included in the replication as to maintain independence. Three of the variants were absent in BRASS, highlighting the difficulty of replicating rare variants in a small number of independent samples. However, rs1800888 in *ADRB2* was present in the BRASS study at similar frequencies (asthma cases = 1.6% vs. controls = 0.49%, p = 0.31).

**Table 6:** Results of replication in the BRASS study of four variants associated with asthma following severe RSV bronchiolitis in infancy (number of asthma cases = 81, number of asthma controls = 126).

Position (hg19)	rsID	Gene	Alleles (A1/A2)	Freq A1 Cases	Freq A1 Controls	P-value
chr1:152283023	NA	*FLG*	T/A	0	0	NA
chr5:148206885	rs1800888	*ADRB2*	T/A	0.016	0.0049	0.31
chr11:113076804	rs35576001	*NCAM1*	A/G	0	0	NA
chr12:117768154	rs76090928	*NOS1*	A/G	0	0	NA

## Discussion

We identified over 700 nonsynonymous variants by sequencing the coding exons of 131 asthma-associated genes in 182 individuals who experienced severe RSV bronchiolitis in infancy. Following this, we genotyped a set of 190 nonsynonymous variants, and performed association testing for physician-diagnosed asthma before the 7^th^ birthday following severe RSV bronchiolitis in infancy. Given the rareness of severe RSV bronchiolitis in the first year of life, we substantially strengthened our power for association testing by including over 6,503 individuals from the Exome Sequencing Project (ESP) as controls. This allowed us to identify four nonsynonymous variants that were significantly associated with asthma following severe RSV bronchiolitis for follow-up studies. All four of the nonsynonymous variants were low frequency or rare in the ESP (MAF < 5%), and were at higher frequency in individuals with asthma following severe RSV bronchiolitis in infancy (MAF from 1–6%).

**Fig 3:**
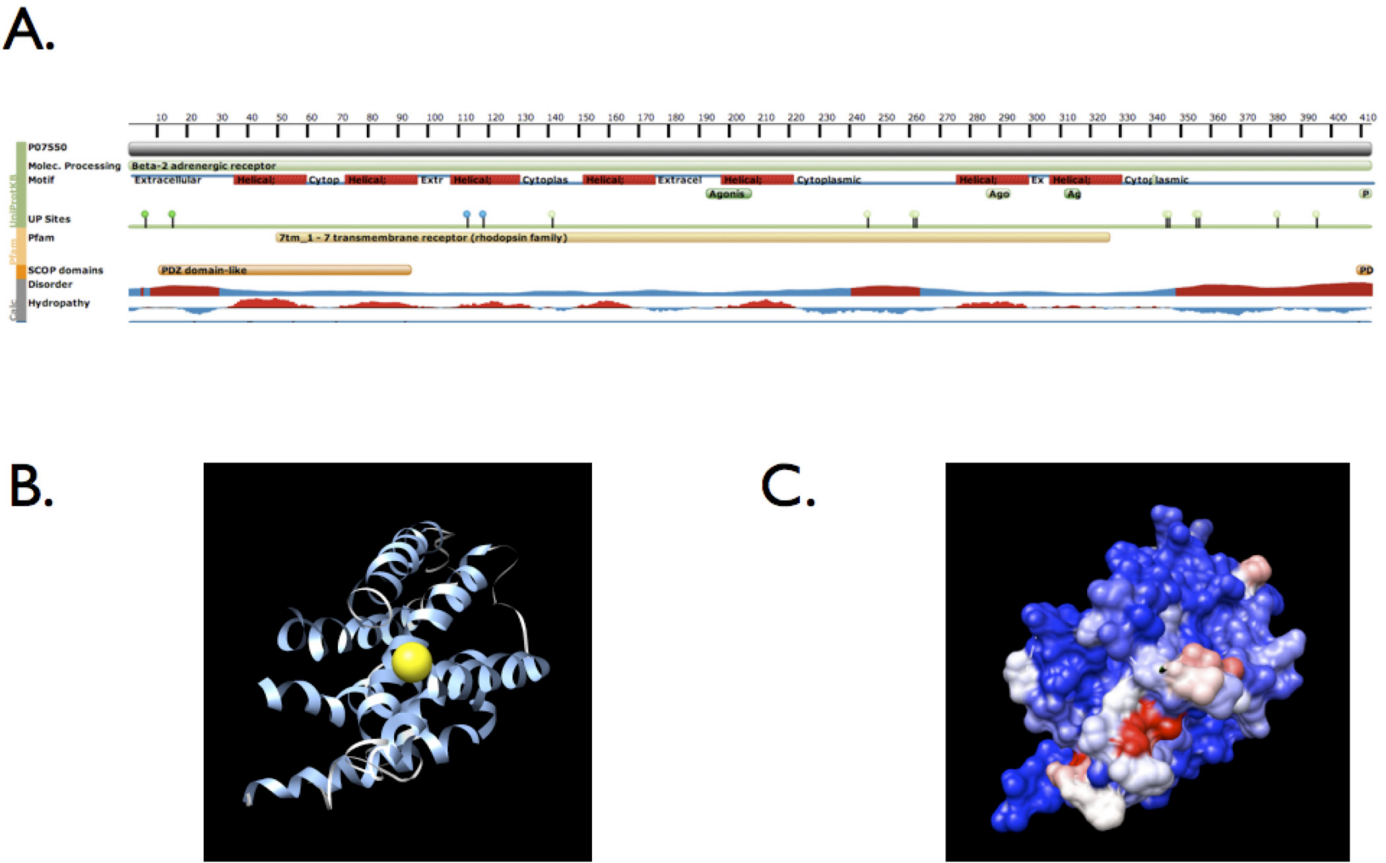
Structural domains and crystal structure of ADRB2 (protein databank [PDR] structure 2r4r, images from LS-SNP/PDB [51]). **(A)** Structural domains of ADRB2; rs1800888 is located in amino acid residue number 164 within a helical transmembrane domain (protein databank [PDB] structure 2r4r) (**B)** crystal structure of ADRB2 showing the location of rs1800888; solvent accessibility for the amino acid residue at rs1800888 is 41% (exposed). **(C)** Position conservation within protein superfamily (G-protein coupled receptor 1 family), red = high conservation, blue = low; 32% of protein sequences in the alignment contain the most frequent amino acid residue at rs1800888.

We identified a nonsynonymous variant in *ADRB2* (rs1800888) that showed a significant association with asthma following severe RSV bronchiolitis in European Americans when using the ESP as controls. Rs1800888 was also nominally associated with asthma and active asthma within individuals with severe RSV bronchiolitis in infancy, without including the ESP as controls. ADRB2 is a G protein-coupled receptor that along with other proteins forms a receptor-channel complex involved in smooth muscle relaxation and bronchodilation. Notably, rs1800888 has the most profound functional consequence of all known nonsynonymous variants in *ADRB2*, and is associated with a 5-fold reduction in sensitivity to beta-2-receptor agonist-mediated vasodilation resulting in increased vasoconstriction.[25] This variant is located in a helical transmembrane domain, and is within an amino acid that is both solvent accessible and in the proximity of amino acids that are conserved across the protein superfamily including ADRB2 (Fig 3). Although rs1800888 has not been previously associated with asthma, other variants within and upstream of *ADRB2* have been associated with both asthma and bronchodilator drug response.[26–30]

We similarly observed an association with asthma following severe RSV bronchiolitis and rare nonsynonymous variants in *FLG* and *NCAM1* in European Americans, and *NOS1* in African Americans. These associations were driven by a single carrier of each variant and thus require additional study. *FLG* encodes the filaggrin protein, which functions in differentiation of the epidermis and maintaining barrier function.[31] Common variants in *FLG* have been associated with atopic dermatitis[32–34], eczema[33,35–38], and asthma.[35,36] NCAM1 is a neural cell adhesion molecule involved in the proliferation of T-cells and dendritic cells, and is a member of the immunoglobulin superfamily. While the function of rs35576001 in *NCAM1* is unknown, it is predicted to be probably damaging by PolyPhen2.[39] NOS1 is a neuronal nitric oxide synthase involved in the synthesis of nitric oxide, which acts as a messenger molecule in several processes including the neural regulation of smooth muscle.[40] Rs76090928 resides within the first exon of *NOS1*, which contains a hypoxia-responsive promotor and is only transcribed in hypoxic conditions.[40] Variants in *NOS1* have been associated with atopy[41,42], total IgE levels[43,44], and asthma.[44–47] However, it is important to note that we did not account for population structure in our African American samples due to the absence of sufficient genetic data to estimate ancestry. And thus, it remains possible that the association at *NOS1* is due to an imbalance in the proportion of European admixture, rather than a true genetic association with asthma following severe RSV.

Given that all of our associations were with low frequency and rare variants, and that a severe form of RSV bronchiolitis in infancy is relatively uncommon (0.5% of RSV bronchiolitis cases in children < 24 months[48]), we were limited in our ability to replicate our findings in an independent study. Further, we were limited in our ability to perform more powerful collapsing methods that combine groups of rare variants for association testing (e.g. SKAT[49]), as data was generated through anonymous pooled sequencing (variants could not be assigned to an individual within a pool). Therefore, additional studies are required to confirm the role of the rare variants we identified in the etiology of asthma, and to identify whether there are additional rare variants that contribute to asthma following severe RSV bronchiolitis in infancy.

It is important to note two limitations of using the ESP as controls in our study. First, given the heterogeneous clinical characteristics of the ESP samples they cannot strictly be considered “population controls” as some individuals may have experienced asthma following severe RSV bronchiolitis in infancy. The goal of the ESP was to identify genetic variation relevant to heart, lung, and blood disorders in well-phenotyped populations, and contains individuals from clinical studies including 191 African Americans with asthma from the Severe Asthma Research Program (SARP). This could have lead to rare variants that contribute to the development of asthma following severe RSV in infancy being more common in the African American ESP as compared to healthy controls, however this would not have lead to false positive associations, and is not expected to have a large effect on our power to detect an association.[50] Furthermore, allele frequencies in the 1000 Genomes Project are similar to that observed in the ESP for the variants we identified (Table 7). Second, different technologies were used to obtain genotypes in the RBEL study (GoldenGate array) vs. the ESP (next-generation sequencing), which could have lead to differences in allele frequencies caused by technical artifacts. However, we are reassured in that rs1800888 is nominally associated with asthma when not including the ESP as controls (but rather asthma controls from within the RBEL study itself), and that all four risk alleles are either absent or less frequent in both the ESP and asthma controls from within the RBEL study, as compared to asthma cases.

**Table 7:** Minor allele frequencies of four nonsynonymous variants associated with asthma following severe RSV bronchiolitis in the ASW, AFR, and EUR populations from the 1000 Genomes Project, and in European (EA) and African Americans (AA) from the Exome Sequencing Project (ESP).

Position (hg19)	rsID	Gene	ASW	AFR	ESP–AA	EUR	ESP—EA
chr1:152283023	NA	*FLG*	NA	NA	0	NA	0.0001
chr5:148206885	rs1800888	*ADRB2*	0	0	0.0032	0.015	0.015
chr11:113076804	rs35576001	*NCAM1*	0.025	0.022	0.027	0	0.0006
chr12:117768154	rs76090928	*NOS1*	0	0	0	0	0.004

In conclusion, we identified a number of novel, potentially functional nonsynonymous variants through the sequencing of anonymous pools of asthma cases and controls following severe RSV bronchiolitis during infancy. Direct genotyping of a subset of these variants identified four nonsynonymous variants associated with increased risk of asthma following severe RSV bronchiolitis, including a highly functional rare variant in *ADRB2*. Additional studies are needed to confirm these associations and determine the functional consequences of these genetic variants.

## Supporting Information

S1 Table List of candidate asthma-associated genes. (DOCX)

S2 Table Forward and Reverse PCR primers for each amplified region of genomic DNA (N = 938). (DOCX)
