# Supplementary material for: Pooled Sequencing of Candidate Genes Implicates Rare Variants in the Development of Asthma Following Severe RSV Bronchiolitis in Infancy
Source: PLoS One. 2015 Nov 20;10(11):e0142649. doi: 10.1371/journal.pone.0142649 (PMC4654486; doi:10.1371/journal.pone.0142649)
Supplement: S1 Table — (DOCX) [file pone.0142649.s001.docx]

**S1 Table.** List of candidate asthma-associated genes.

| **Gene Symbol** | **Full Gene Name** |
| --- | --- |
| ABCC1 | ATP-binding cassette, subfamily C member 1(Also known as Multidrug resistance-associated protein 1; MRP1) |
| ADAM33 | A Disintegrin And Metalloprotease 33 |
| ADCY9 | Adenylate Cyclase 9 |
| ADRB2 | Beta-2 Adrenergic Receptor |
| ALOX5 | Arachidonate 5-lipoxygenase |
| ALOX5AP | Arachidonate 5-Lipoxygenase Activating Protein |
| ANXA1 | Annexin A1 |
| AQP1 | Aquaporin 1 |
| ARG1 | Arginase-1 |
| ARG2 | Arginase-2 |
| AURKB | Aurora Kinase B |
| BMP1 | Bone Morphogenetic Protein 1 |
| BMP6 | Bone Morphogenetic Protein 6 |
| C11orf71 | Chromosome 11 open reading frame 71 |
| CAT | Catalase |
| CCL11 | Chemokine [C-C motif] ligand 11 |
| CCL2 | Chemokine (C-C)Ligand 2 ( MCP1) |
| CCL24 | Chemokine [C-C motif] ligand 24 |
| CCL5 | Chemokine CC Motif Ligand 5 (RANTES) |
| CD14 | CD14 molecule |
| CFL1 | Cofilin 1 |
| CFTR | Cystic fibrosis transmembrane conductance regulator (ATP-binding cassette subfamily C, member 7) |
| CHI3L1 | Chitinase 3-like 1 protein |
| CLCA1 | CLCA family member 1, chloride channel |
| CRCT1 | Cysteine-rich C-terminal 1 |
| CSF2 | Colony Stimulating Factor 2 |
| CTLA4 | Cytotoxic T-lymphocyte-associated protein 4 |
| CX3CR1 | Chemokine [C-X3-C motif] receptor 1 |
| CXCL5 | Chemoking CXC Ligand 5 |
| CYFIP2 | Cytoplasmic FMR1 interacting protein 2 |
| CYSLTR1 | Cysteinyl leukotriene receptor 1 |
| CYSLTR2 | Cysteinyl leukotriene receptor 2 |
| DPP10 | Dipeptidyl-peptidase 10 |
| ECE1 | Endothelin Converting Enzyme 1 |
| EDN1 | Endothelin 1 |
| EPHA5 | EPH receptor A5 |
| ERG | Erythroblastosis virus E26 v-ets oncogene homolog |
| ESR1 | estrogen receptor 1 |
| FBXO43 | F-box protein 43 |
| FLG | Filaggrin |
| GALNT10 | Polypeptide N-acetylgalactosaminyltransferase 10 |
| GFAP | Glial Fibrillary Acidic Protein |
| GSDMB | Gasdermin B |
| GSTM1 | Glutathione S-transferase M1 |
| GSTP1 | Glutathione S-transferase pi |
| HAVCR1 | Hepatitis A virus cellular receptor 1 |
| HBB | Hemoglobin beta |
| HLA-DRB1 | Major histocompatibility complex, class II, DR beta1 |
| HLA-G | HLA-G histocompatibility antigen, class I, G |
| HMOX1 | HemeOxygenase1 |
| ICAM2 | Intercellular adhesion molecule 2 |
| IFNg | Interferon gamma |
| IGFBP6 | Insulin like growth factor binding protein 6 |
| IKZF3 | IKAROS family zinc finger 3(Aiolos) |
| IL10 | Interleukin 10 |
| IL13 | Interleukin 13 |
| IL1RAPL1 | Interleukin 1 receptor associated protein-like 1 |
| IL1RL1 | Interleukin 1 receptor-like 1 |
| IL33 | Interleukin 33 |
| IL3RB | interleukin 3 receptor/GMCSF 3 receptor, beta |
| IL4 | Interleukin 4 |
| IL4R | Interleukin 4 Receptor |
| INPP4A | Inosytol polyphosphate-4-phosphatase, type 1 |
| IRAK3 | Interleukin-1 receptorassociated kinase 3 |
| ITGAV | Integrin Alpha V |
| ITGB3 | Integrin, β-3 |
| KL | Klotho |
| KNG1 | Kininogen 1 |
| LCN2 | Lipocalin 2 |
| LMNA | Lamin A/C |
| LRP1B | Low density lipoprotein receptor-related protein 1B |
| LSP1 | Lymphocyte specific protein 1 |
| LTA | Lymphotoxin Alpha |
| LTA4H | Leukotriene A4 hydrolase |
| LTC4S | leukotriene C4 synthase |
| MET | Met Oncoprotein |
| MPO | Myeloperoxidase |
| MS4A2 | Fc fragment of IgE, high affinity I, receptor for; beta polypeptide |
| MYLK | Myosin Light Chain Kinase |
| NAT2 | N-acetyltransferase 2 |
| NCAM1 | Neural cell adhesion molecule |
| NOD1 | Nucleotide-binding oligomerization domain containing 1 |
| NOS1 | Nitric oxide synthase 1 |
| NOS2A | Nitric oxide synthase 2A |
| NOS3 | Nitric oxide synthase 3 (endothelial cell) |
| NPPA | Natriuretic peptide precursor A |
| NPSR1 | Neuropeptide S receptor 1, also know as G protein-coupled receptor 154 (GPR154) |
| ORMDL3 | ORM1-like 3 |
| PER1 | Period circadian protein homolog 1 |
| PHF11 | PHD finger protein 11 |
| PLA2G4A | Phospholipase A2, group IVA (cytosolic calcium-dependent) |
| PLA2G7 | also called,PAFAH; platelet activating factor acetylhydrolase, (Phospholipase A2, group 7) |
| PLAU | Plasminogen activator, urokinase, also known as UKTPA |
| PPBP | Pro-platelet basic protein |
| PPIB | Peptidyl Prolyl Isomerase B |
| PTGDR | Prostaglandin D2 receptor (DP) |
| PYHIN1 | Pyrin and HIN domain family, member 1 |
| RASSF8 | Ras association (RalGDS/AF-6) domain family (N-terminal) member 8 |
| RCAN2 | Regulator of calcineurin 2 |
| RCBTB1 | RCC1 and BTB domain containing protein 1 |
| RTP2 | Receptor (chemosensory) transporter protein 2 |
| RUNX1 | Runt related transcription Factor 1 |
| RUNX3 | Runt-related transcription factor 3 |
| SCGB1A1 | Secretoglobin, family 1A, member 1 |
| SELL | Selectin L |
| SELP | Selectin P |
| SENP6 | Sentrin specific peptidase 6 |
| SERPINB3 | Serine peptidase Inhibitor, clade B member 3 |
| SETDB2 | SET domain, bifurcated 2 |
| SLAIN1 | SLAIN motif family, member 1 |
| SPINK5 | Serine peptidase inhibitor, Kazal type 5 |
| SRP9 | Signal recognition particle 9kDa |
| STAT6 | Signal transducer and activator of transcription 6 |
| TBX21 | T-box 21 |
| TBXA2R | Thromboxane A2 receptor |
| TEK | Tyrosine Kinase, endothelial |
| TGFB1 | Transforming growth factor, beta 1 |
| TGFBR2 | Transforming growth factor beta receptor II |
| TGFBR3 | Transforming growth factor beta receptor III |
| TLR4 | Toll Like Receptor 4 |
| TLR9 | Toll-like receptor 9 |
| TNF | Tumor necrosis factor |
| TNIP3 | TNFAIP3 interacting protein 1 |
| TRAP1 | TNF receptor associated protein |
| TSLP | Thymic stromal lymphopoietin |
| UBC | Ubiquitin C |
| URI1 | URI1, prefoldin-like chaperone |
| VCAM1 | Vascular cell adhesion molecule 1 |
| WDR36 | WD repeat domain 36 |
| ZPBP2 | Zona pellucida binding protein 2 |
