# Supplementary material for: Pooled Sequencing of Candidate Genes Implicates Rare Variants in the Development of Asthma Following Severe RSV Bronchiolitis in Infancy
Source: PLoS One. 2015 Nov 20;10(11):e0142649. doi: 10.1371/journal.pone.0142649 (PMC4654486; doi:10.1371/journal.pone.0142649)
Supplement: S2 Table — (DOCX) [file pone.0142649.s002.docx]

**S2 Table:** Forward and Reverse PCR primers for each amplified region of genomic DNA (N=938).

| **amplicon** | **Forward Primer** | **Reverse Primer** |
| --- | --- | --- |
| ADAM33c12c16_F1 | ACCAAAGAAACGCAGCGGAGGAAGT | AGCTTCCACTCAGGGTCCCCATTTG |
| ADAM33c17c19_F1 | CCCGTCTCTTCTGATTTGGAGGGCT | GCATTTAGGGAGGAAGGCTGGGAGA |
| ADAM33c20_F1 | CTCCCTGTCATCTGCACCCTCTCTG | GTTCTCCCCAAGAACTTCCTCCCCA |
| ADAM33c21_F1 | AGTGCCTTCTTCTGCCCACTTGAGG | TGTTGAAGGTGAAGTCTGCCCCAAA |
| ADAM33c22u22_F1 | AAGAGTCCCCACGCCCTGACCTACT | TTCCCAGCGGTTCTCTCCTCCTCTC |
| ADAM33c2c3_F1 | CAGAAACCTGATTAGGGGGCAGCAA | TCACTGTGCCAACCTCCTGGACTCT |
| ADAM33c4c8_F1 | AGTAGGCTCAGGAAGCAGGCGCTC | TTCTACTGTGGGGAAGATGGGCAGG |
| ADAM33c9c11_F1 | AGAAAACTGGCCCCACCAGAGGATG | GGCGAGGTTACTCCTACACCGGGAG |
| ADAM33u1c1_F1 | AAACTAGGCAACACCGTCCTGTGCC | GCCCCAGTCCTTAGGGGACAACATA |
| ADCY9c10d1_F1 | AGTCACACTGACCCTGAGATCCCCC | TTTGGCTACCATTTCCGGGATGAAG |
| ADCY9c1d4_F1 | CCAAATCGACCCCTTTCTTCGGCTT | CCTTTCCCGTGTCTTTGCTCTCCAG |
| ADCY9c2_F1 | TTTCAGGAAGCTTCGAAATGGGCAG | AAGCTCTTCTCAGGTTGCCTTGTGG |
| ADCY9c3_F1 | AGAAGCAATGTGCAAATGCAGCCAG | TGTCCTAACCCTTCGCCTCTTGCAG |
| ADCY9c4_F1 | TAGGGAGTCCAGGAGAGCGTGGAAA | CTCCTGCTAACCCCCATGCCTCTC |
| ADCY9c5_F1 | GCTCAGAGCTGGAGACCCAGTCCAC | TCATTCTGCTCCACTCCTGCTCCAC |
| ADCY9c6_F1 | TGTTCCCATCCCTTGGTAAAGCACA | CATTGGCACAGTTATGGTGCTGGGT |
| ADCY9c7_F1 | TTTTTCCCAGACAGCCCCAGTCGTA | CTTCCCGTGTCTGCGTCCTCTTTGT |
| ADCY9c8_F1 | CGCCAGGAGAGGAACTGACAGGAAC | GGTTTCTGGGTGTGTCCCCACAGTC |
| ADCY9c9_F1 | TGCCTCAGACCTGGAGGCTGCTTAT | CTGCGTTTCAACAGACGGTGCTTTC |
| ADCY9cu10d2_F1 | ACCTCACCTGGGACATGACGAACAG | GCTAACTCTCCCCGTTTTGCTGGC |
| ADCY9u11_F1 | ATCTGTTCCTGTGGTTCCCGGCTC | CCGAGTGGGAAGGCAACATTTTGAT |
| ADCY9u1d1_F1 | CCCTCCTCTCTGAAGCAGTGCTCATC | TTTGAATGTCCTCCCCTCCCCTTTC |
| ADCY9u1d2_F1 | CGAGACAGCAAGGTCGTGAGCAAAC | GGAAGCTGACTCATCGGAGAGGGAA |
| ADCY9uc1d3_F1 | CCAGGTGGCCAAAACACAAAAGAGA | ATCTCTGGGTTCTGACAGCAGCACG |
| ADRB2cu1d2_F1 | CCAGAACCTTAGCCAGGTGGAGCAG | AGGGGTTTTGGAGAAACAGGTGCAA |
| ADRB2uc1d1_F1 | CGAGGGCAGTTCCCCTAAAGTCCTG | TTGAGGGCTTTGTGCTCCTTCAAGC |
| ANXA1c10_F1 | TTTGCTGTGGCTCTTAGAACTGCTG | GAACATAGGTTCCCCAACCCTGATGG |
| ANXA1c11_F1 | TGGCTTCTCCACTCCCAAGGAGAAT | CCCGTGGTGACACATGTTTTAAGCAA |
| ANXA1c12_F1 | CCGGATGGTGAGAGATGAGTTAGGA | TCCTCCACAAAGAGCCACCAGGATT |
| ANXA1c13u13_F1 | CCTTTGCCAAGCCATCCTGGTATGT | ACCATGGCTGATGCTCTCCTTGAAA |
| ANXA1c4_F1 | GCAACACTGAGGGCCAGGAAAAGTT | CCAGAGCACCACATCTGCAAACAAT |
| ANXA1c5_F1 | CAGATGTGGTGCTCTGGGGACAAAT | TGTCAGCAACCTAGCAACTCAAGCG |
| ANXA1c6_F1 | CGCCCAATTTGAGCAAACTCCTTTC | TGTCAGCAACCTAGCAACTCAAGCG |
| ANXA1c7_F1 | GGCTTTTTGGCTTTCCCAATGCAA | CGGTCACCCTGAAAGAAATCCCATT |
| ANXA1c8_F1 | TCGGAACGCTTTGCTTTCTCTTGCT | CAGGGGATCATGTGGTAGCCCC |
| ANXA1c9_F1 | TTGGGCACAAAGCGATTGCCTAT | GTTCCACATGCCCAAGTCACAAAATG |
| ANXA1u1_F1 | CCATGTACTTCAAACAGAAGGCAGCCA | AAAAGCAGCCCCCATCACGATTTCT |
| ANXA1u2c3_F1 | TGATCCTGGAAAGTAAGCGCAAGGC | TCCAGGAGGGAACTGTTTTGAGGAA |
| ARG1c2_F1 | CTGTTGCCTGCTTTCTCCCCAAGAG | AGGTTCACAGGGGCCTGCTCTTTC |
| ARG1c3_F1 | TGGCATTCCCTATTAAGCCCAAACCA | CCTTTGTGCCAGATTCCCATAGACACA |
| ARG1c4_F1 | ATGAGTGAACACACGCCATACGCTG | TCATTTCCCTTCCACCTCCTGAATG |
| ARG1c5_F1 | CAAGGGGAGGCACACATCCTCTTCT | GACTGTGCCCGCCTGTCTCCTTTTA |
| ARG1c6c7_F1 | CAGGCGGGCACAGTCAGCCTTATTA | GCTGGGACTTGGTTTCAGGTCAAGG |
| ARG1c8u8_F1 | GACCTGAAACCAAGTCCCAGCTGAC | GCCCAATGAACTTGTAGATTGCCCA |
| ARG1u1c1_F1 | ATGTGACAGACGATCTTGCCAAGCC | GGCTGAGATCACGAGCCACAAAAGT |
| BMP1c10c12_F1 | GGGTTCACCACTCTTCCATCCACAC | GCAGGGGAGAGTAACAGAGGAGGCA |
| BMP1c13_F1 | TCTGTGACACCCTTTCCTTCCCACC | AGAACAGGGTCAGAGGCAGTCTGGG |
| BMP1c14_F1 | CCTTACTGCATCCCAGCCCACATCT | GACAGGAAGCAGGCACTGAGCTGAA |
| BMP1c15_F1 | CCCGAGGGCAGGAGTAGTCAAGAGA | GATGGGAGAGTGAAGTGGTGGGGAG |
| BMP1c16_F1 | TCTCTTCCTGCCCTTCTGTTGCTCC | GGGGAAAGTGAGTCCTGAGGAAGGC |
| BMP1c17_F1 | CACCCCTTGGTCCCTTTTCTCCTTC | GGACCAGCATGGGTCACCATTACCT |
| BMP1c18_F1 | AGGTAATGGTGACCCATGCTGGTCC | TGGAGATGATCCTCTGGGCTTCCAC |
| BMP1c19_F1 | GGTCCCTCTCTATCCCCTTCCGTCA | GACAGTGAAGGAGGGCCCAGGAATA |
| BMP1c2_F1 | GCACCTAGGGCTGGGTAGGAGGATT | AAACTCATGGCCTCTGTCCTGGGAG |
| BMP1c20_F1 | TCTGTGCTCCTTCCTCATCCCCCTA | AACACTCCCACAACCGGGGACAGTA |
| BMP1c21_F1 | TAGAGATGGGGCAGCACAGCAATGT | CTGAGAAGGGTGGAGGACAGTGTGG |
| BMP1c22u22_F1 | CCCTCTTCCTCCACCTTACCCCATC | CCACAGCAAGCCCATACATCTGGAA |
| BMP1c3c4_F1 | GGTATGGCACAACGGAAAGCTGCAT | GTACAGCGCCTAGGGCTTCCTGCTC |
| BMP1c5_F1 | TATGGGGCATCTACCCAGGAACTGC | GACACGGAGGAGGAAATGCCTCAAG |
| BMP1c6_F1 | GACTCCATTCCCTGCCCTGGAAAGT | GTGGGAGGCAGGAGGAGAATAGCCT |
| BMP1c7_F1 | CACTGTCCATGAGACGCTCACCCTT | CTTGCAGCCTAAGCCTTCTTGGTGG |
| BMP1c8_F1 | TGCCCTGTCATTTCCTTTCCTCACA | CCACTGTGGGAGAAGTCCCTGAGGT |
| BMP1c9_F1 | TTAACCCTCTTCCTCCCCTGTTGCC | GGTGGCTCAGAGAGGGAATCAGCAT |
| BMP1u1c1_F1 | CGAGCTCATTTCCCTAAAAAGGGGG | CTCTCCCCCTCGTTCTTTTGTTCCC |
| BMP6c2_F1 | GGTGAGGTAAGCCCGTGGCACTTAG | TTTGGGGCTTTCTCGAGCCTTCATA |
| BMP6c3_F1 | GCAGTGCGCTATTTACCAGGCCATT | ATGACTTGCTGGACGTTGGAGGCTT |
| BMP6c4_F1 | CCAGGCAAGGTTTGGGGATACATGA | CTTTCTGGAAACACAGGCTCCCCAC |
| BMP6c5_F1 | CTGACCCCAGGGTAGACAAAACGGA | GCTCTGGGTGTTTGGTAATGGCTGC |
| BMP6c6_F1 | TATGCAGGTGGCACTACTTCCGCAG | CACAGCACGGTTTGGGGACATACTC |
| BMP6cu7d1_F1 | CAACGCACACATGAATGCAACCAAC | CACCCCTTGTAGCCTCACCTCAGTG |
| BMP6u1c1_F1 | GTAAACTTCATGGTGGCCCTGCGAT | CGGGACACTGAAAAGGGGGAAATGT |
| BMP6u7d2_F1 | TCGTGCTGGAGTTTTGTTGGTGTGA | AACAGCAGCATCTCGACCAGCCTCT |
| CATc10_F1 | GCAGATGGCAGCGTTCCCTAAGAAT | GGGGGATTATGCAGGCATTGACAAG |
| CATc11_F1 | TCCTCCATGGTTCTGGGAGAATTGA | TAGCCAGCAGAGGACACCCTTCAGC |
| CATc12_F1 | AACACTGGGAAACCACAGTCCCTGG | TGGCCCCTGCAGACTGTAATGCTAA |
| CATc13u13_F1 | CGTTGCTGCCCATGAGGTGATTAAC | TGGAGTCCTCGAGATACTGGCATTTCC |
| CATc2_F1 | GCAATGGCCCATCCTGTCAGATTTT | CACTTGACCCAGGTGCTGTGAAACA |
| CATc3_F1 | CAGGTGCCTGTTGAGGACCTGAATG | GCCATACCAATCACGCCAATAAACCA |
| CATc4_F1 | GCTGTGGCTTATGCTTCCTGTTTCCA | CGGCTTCTCCTCAAATGCCAACCTA |
| CATc5_F1 | GCATGCGGGAGGGTGGGTATATTTT | GGCCGATGAATGGGGGATGATTAGT |
| CATc6_F1 | GGCCAGTTTTCAGGTTGTTTTGGCT | GAGCCTTGTACCCTCTGCCCCAAAG |
| CATc7_F1 | TGGCCGAGCACAGTCTCAGTAATTG | TGAGAGGGTAGTCCTTGTGAGGCCA |
| CATc8_F1 | CATTGCCACAGGAAAGTACCCCTCC | CAGAACATCTCAATCCACCAGGCTCA |
| CATc9_F1 | GCGGGAAAGGCAGAATTTTGTGGTA | GGGAGCACCTTTACCAAGCAGGAGG |
| CATu1c1_F1 | GTGCTGATTGGCTGAGCCTGAAGTC | GTAAAAGTCCGTCTGCACCGAAGGG |
| CCL11c2u3_F1 | CCACCTCTCGCAATTCCTTGCTTTC | ACCCCCTTAACCAAGCCCCTCTTG |
| CCL11u1c1_F1 | TGGTCAGCAATCAGGAATCCCTTCA | GAGCCATTCCCCATTCCCAAACACT |
| CCL24c1_F1 | CAAGCAGCTCAGGCCCAAACTCAG | ACCTCAGCTGATCCCCTGTCCCTTT |
| CCL24c2c3_F1 | GCCACGTGCCCATGAAGGATACCTA | TTTCCTCCTGCACGTCAGCTTTGAG |
| CCL5c2_F1 | ACTCCTCAGGGAACAGGCTCTGGG | AATGAAGCCAGAGGACCCAGGACAA |
| CCL5c3u3_F1 | CAGTCATTGGGATGGGGTAGGCATT | GGATGCCCCTCAACTGGCCCTATAA |
| CCL5u1c1_F1 | GTTCCCCGGGAGGAAATCAAGAGTC | CCTCCCTCTTCTTCTCCTCTCCCACA |
| CD14c1d2_F1 | TAGATTCTGGATGGCCGGGAACTTG | ATGGGGCCTCCTAGACCTCTGCTCT |
| CD14c2u3_F1 | CGTTCGACCCCAAGACCCTACACTC | GCAACGTGCAAGTGGGAAAACTGAT |
| CD14uc1d1_F1 | CCCGCAGTTCTTTTCTTGAGGAGGA | GCCAGGCCTCAAGGTACTGAGCATT |
| CFTRc10_F1 | TCAGAGGAGGGTGCTACCAGTGTGA | CATGATTTTCCTGGACCAGCTCTGA |
| CFTRc11_F1 | GCAAGTGAATCCTGAGCGTGATTTGA | GCTGGCAGATCAATGCTCATTCCATT |
| CFTRc12_F1 | AGGTGCTGAGGATGGACGAGGAAGA | ACCCACTAGCCATAAAACCCCAGGA |
| CFTRc13_F1 | GTGCTACTTCTGCACCACTTTTGAGA | CTGGCCCAGTAGGGCAGATCAGATT |
| CFTRc14_F1 | GGCTGGGTTATTCCTAGAAGTACCTTTC | GGTGAAATACCCCCAAGCGATGTAT |
| CFTRc15_F1 | AAATGAGCCATGGGCAGGGTTGTAA | TGCTTTGAGTGTTTTTGTTGTTGCTG |
| CFTRc16_F1 | GGACCCAGGAACACAAAGCAAAGGA | CAGGAGCTGCACATGCTCACAATTT |
| CFTRc17_F1 | GCATGTGCAGCTCCTGCAGTTTCTA | GGCCCTATTGATGGTGGATCAGCAG |
| CFTRc18_F1 | GAGCAGCCAGGGTTTCGGTAGAGGT | CAGGTTTGGGCCAGGTAAGCAGTTC |
| CFTRc19_F1 | CGGGTAGAGAACTGGCTCACTGAACA | GCAGCTCCAACACATGAAGAAAGGG |
| CFTRc2_F1 | GGTGTAGCCTGTAAGAGATGAAGCCTGG | AACCAGCATAAGGCAAATGCCACCA |
| CFTRc20_F1 | TCCCAGTGGTAGCCAAGAAAGAGGA | GCAAATGCCTGCTTTCGGTTTGAGT |
| CFTRc21_F1 | CCTACCCATTACCAACAACACCTCCA | AATGGGATTTGTGCCCCTGATATGG |
| CFTRc22_F1 | CCTGTTAGTTCATTGAAAAGCCCGAC | GCCCAGTGCTGGGATCCATAAGAAG |
| CFTRc23_F1 | GGGGTCCAATTCCTTATGGCCAGTT | CAAAAGCCCAAGGCTCCCACTGTAA |
| CFTRc24_F2 | TGAGGCTCTCTCTTGTGCCTGTCTGA | CGCGCCAGGTATTTTAGGGTACTCCAT |
| CFTRc25_F1 | GGCAGGTAGTGGGGGTAGAGGGATT | CCACACGCAGACATGACAGCCTAAT |
| CFTRc26_F1 | CCATGGTTGAAAAGCTGATTGTGGCT | CAACCTCACCACATGGCTCAGATCA |
| CFTRc3_F1 | TGCACATGCAACTTATTGGTCCCAC | CCACCCGTGTTCCAGGACATTTTAG |
| CFTRc4_F1 | CCTCCCATTCCCAGCTCTGCTTTGT | CATGGGGCCTGTGCAAGGAAGTATT |
| CFTRc5_F1 | GCTCAGTGGATTTCCCCCACTTCAT | CCAGGAAAACTCCGCCTTTCCAGTT |
| CFTRc6_F1 | CCCAACTCAAAGGCACCTTAGCCTG | GCAGTCCTGGTTTTACTAAAGTGGGC |
| CFTRc7_F1 | GGCTGGGCTAGGGAGAATGATGATG | CAGCCCATGAAAGTGAATTTGTGC |
| CFTRc8_F1 | TGCTGGAGCACTGAAGCCTCACATA | GCCACTCTCATCCATCATACTGTCCA |
| CFTRc9_F1 | TCCGTCCAATGTTGGATTAAGGCAC | CTCTCGCATCCTTTTATTGGCTGGT |
| CFTRcu27d1_F1 | TGCTCTGGTCTGACCTGCCTTCTGT | AAGGGAACCATCCTGTCCCCTGTG |
| CFTRu1c1_F1 | AAGGAAGGGGTGGTGTGCGGAGTAG | ATACACACGCCCTCCTCTTTCGTGG |
| CFTRu27d2_F1 | ACCACAGGAACCACAAGACTGCACA | CATGCAGGTGCCAACGCAGAAATAG |
| CHI3L1c10u10_F1 | TCTGGGCCTGAAAACCCACAACTCT | CTTGCCAGCCTCGTGATTCCTTCAT |
| CHI3L1c3_F1 | GGGGGAATCCTACCTTCTCCCCAAC | CAACCTTCCGCACCTGTGCACTAAC |
| CHI3L1c4_F1 | AATGACTCCTACCTCCTCGGCTCCC | TCGTTCACATCCTGCTGGAGCCTTA |
| CHI3L1c5_F1 | GAGTGTTGGCAGAGGTTCTGGGGAG | ATGCCACTTGGGACCCTTTCTCTCA |
| CHI3L1c6_F1 | CTTCTAGCCCACCCCATTCCCTCAT | GAGGCCCAAGGCACCTAGCATATCA |
| CHI3L1c7_F1 | ATACAGTGGATGCGGGAGACCCAAG | CATGAAGCCTGTGACCCCTCAGAGA |
| CHI3L1c8_F1 | GTCATCCCCTCCACCCATTTAAGCC | AGCCTGAGGTCTCTTGCCGAATCCT |
| CHI3L1c9_F1 | TAAGACCTCCTCCACACCTGCCCTG | ATCTGCCTGCTGGGTGAGGGACTAA |
| CHI3L1u1c2_F1 | GGGCGCTCAAAGATAGACCAAAGCA | TCCCCACACTGCCTTTTCCCTGTTA |
| CSF2e3_F1 | CACCGACGAACGACATTTTCCACA | TAAGACATTCCTGCTGGAGGCCCTG |
| CSF2e4u4_F1 | CACGTTACCCACTTGCCTGGACTCA | AGTGTTGGCCCTTGGATAGTGCCC |
| CSF2RBe10_F1 | GGGTATGGCAGGAGCTAGGAGCCAG | GCATTGGGGATTTTCTGGCAGTGAG |
| CSF2RBe11e12_F1 | GACATTCCTCTTTCTCCCCGGCTG | AGAGAGGCAGGAACAGAGAAGCCCC |
| CSF2RBe13_F1 | ACCGGCCCAAATGTCTCTGCTCTT | AACCCCAAAGGTCAGGAGTGATCCA |
| CSF2RBe14_F1 | CCAACTCTTCTGCCCATTTTCTTCCC | GAAATGACTGAGGAAGGTCAGGCGG |
| CSF2RBe3_F1 | TGACCCCCTTCTACCCCTCTTGTCA | CTCTCTACACTGTCGCCCCATGCC |
| CSF2RBe4_F1 | GCTAAGCCGTGTCCTCTCCCAACA | GTCCACACAGGAAACCAAGCCCC |
| CSF2RBe5e6_F1 | TAGGTGCCCTTCACTTCCTCCCCTC | CATTTCCTGGGCTCTGGCAACATTA |
| CSF2RBe7e8_F1 | TGGGAGGGATGAATGACGGAGTACA | CACAGCGGGCTAGGAGCAAACTCAC |
| CSF2RBe9_F1 | CTCCTGAAAGCTTCCCTCCCTCCA | GGAATGCCTCTGGTTGCTTTCCCTT |
| CSF2RBu1_F1 | CGGCACTGCTTCCTCTTTCTGCTTC | TTTCATCCTGGACTCCCCACATTCC |
| CSF2RBu2e2_F1 | GGCAGCTCACTGCTGACATCTCCTT | AGTGAGGACAGGGACAGGGAAGTGG |
| CSF2u1e2_F1 | AGAGCTCTTTTGCCAGTGAGCCCAG | GCAGGGGCTTCTGGAAAGCTATGTC |
| CTLA4c2_F1 | TGAGTTCCCTTTGGCTTTTCCATGC | GCCACCCACAATAAGCAAGGCTACC |
| CTLA4c3_F1 | TCACCAATGTTGGGGAGTAGAGCCC | GCCAATGCCTACGGTTCTAGTGCGT |
| CTLA4cu4d1_F1 | GCTGTAGGTTCAGCAGTGGGGATGA | ACCCAAGGTGGAAAGACACTGCCAT |
| CTLA4u1c1_F1 | GCTAAACCCACGGCTTCCTTTCTCG | CACCTCCTCCATCTTCATGCTCCAA |
| CTLA4u4d2_F1 | GGCAGGGAGCGAGGGAGAAGACTAT | AATGGCTGGTGCAAAGCTGACTCAT |
| CX3CR1cu1d3_F1 | GGAGCAATGCATCTCCATCACTCGT | CCCTTTCTCATCCACACATTGGTCA |
| CX3CR1u1d1_F3 | CGCACAGAGTTTTCTCACATTGTGGC | ATACGTCCACCATGGGGTCCTACCA |
| CX3CR1u2_F1 | GGCTACAACATCCCCAGGAAAATGG | TATCTGAGCTTGCCTGTGGCAGCAT |
| CX3CR1uc1d2_F3 | CCCTTCTTATTTGGGGGCAGGGTTT | TGCATTTGCTGGGGAGAAGTTCAGA |
| CXCL5c2u4_F1 | CCCAGGACAATCCAGAAAAACTTTCCC | AACCTCTTCTTTCCACACTGCCCCA |
| CXCL5u1d1_F1 | TGAGCCAAGGAAATGGTATTGGGGA | CGACATCCTCGAGGCCCTAGC |
| CXCL5uc1d2_F1 | AAGCTGTGAGTGGGGAGGGAACTTG | CGCTGAGTTTTGACAAGGGTGGAGT |
| CYFIP2c10_F1 | CTGTATTGTGCCCCACCCTGGGATA | ACATCTTGGCGCTGGTAACCTCAGC |
| CYFIP2c11_F1 | ATCGGAAGCAGAAACTAGGGGTCGG | TGTGTTCTGATGGAATCCACCTGCC |
| CYFIP2c12_F1 | GGGACCCTCTCCGACCCCATAATTT | TTCCTGGCTGCTCCTTACCCCTTCT |
| CYFIP2c13_F1 | TGAGTATGACACCTGATGCTCCCCA | TGAGGCCCTCTACCCAGAGACACCT |
| CYFIP2c14_F1 | GGGCCTCACACTGACCTCTCTTTCC | GAGGGAGATGCAGGGAGTCTGCAC |
| CYFIP2c15_F1 | GAACATGACCCTTGGCCTCTTCCTG | GCTCCTGAAGGCATCTGAAAACCCA |
| CYFIP2c16_F1 | ACACCACGGGCCCTAGAAACACAAG | ATAAGACCGGAGCAGCCACTCAACC |
| CYFIP2c17_F1 | CAACATGAAGCAACCTTTTTCCCCG | CTCATCCCCTCCTCCCATGCCTTAG |
| CYFIP2c18_F1 | AGCCCACAACCACTGCCTCTCAAG | GCCCTCTACTTGTACCCCAGGAGCC |
| CYFIP2c19_F1 | CGGTGGAGGCTCAAGGAACGTAAGA | GCCCTCAGAGGTTACACAGGCCTTC |
| CYFIP2c20_F1 | GCCTGACTGCTGTCTTTCAGTTGGC | TGAAACCCAAGAACTCACGAGGGTC |
| CYFIP2c21_F1 | AGTGGCTCAACTTCTGTGTTGCCCA | CCAAGGTGGCTTCTGCTCCCCATAC |
| CYFIP2c22_F1 | CCTGAACCTCTTCCCACAGCCTGA | AGAAATCCCACACGCTCCCAGACTC |
| CYFIP2c23_F1 | GCTGTCACTTGCGTGAACAGAAGCA | CTGGGCCTAGTTTGGAGGAGGCAG |
| CYFIP2c24_F1 | TGGCCAGGAAGCACTCCTGTTATCTG | CAGACTTTTGCCCCCATTCCTGCTA |
| CYFIP2c25_F1 | CCAGGACTCCACCAGTGTCCATTCA | TCAAGCCACACTGAGGCCTTTCCTT |
| CYFIP2c26_F1 | ACTGTTCCCCGCCTTTAGGCAAGAG | GGCGGGGTAGGATGGATAGCCAGTA |
| CYFIP2c27_F1 | GCTGCCACTCAGTCATTGTTTCCCA | CCCACTTCCTTTGGGCTCTGGACTT |
| CYFIP2c28_F1 | AGCCACCTGCCTTGAGCCTACAGAC | CCAAAGCTTTAACCTTCCCCCTTGC |
| CYFIP2c29_F1 | CTCAGGAGGAGGTCTGCGATTTGCT | GCAGACGACTTGTTGGCAGGATACA |
| CYFIP2c30_F1 | TGGCAGATGGGCTCTAAGATGTGGA | GTTGGGGGTAACCCTGCCTTCTCTG |
| CYFIP2c31_F1 | GCTGTACTCCTGGCCCAAGATGAGG | CTGGTAAAAGCCTGGTTGTCAGCCC |
| CYFIP2c4_F1 | GTTTGTTGGCAGCAGTTTGCCCTCT | CACATGCCTCTGGTGTGAGAAGCTG |
| CYFIP2c5_F1 | TTTGGGAAAATGTGGCCTGAGGATG | TCTCTGGTTTGGGGATGAAGTAGGGG |
| CYFIP2c6_F1 | GCAGGGGCTACCCAAGTCTCCTCAC | GGGGCCTTTTCAGTTCTCCAGATGC |
| CYFIP2c7_F1 | TGGACCTTACTCACTGCCCCTCTGC | TCTCTTCCTCCACTTGACAGGCAGC |
| CYFIP2c8_F1 | ACTCTGTGTTAGGTGGGCATGCGAG | ACGAGCATGACAACTGGAGAGGGTG |
| CYFIP2c9_F1 | TACATGCGCTGCCTAACCTGAGGG | TGAGAGGGTAAGCCCAGGCTCCAT |
| CYFIP2cu32d1_F1 | TGCCCTAACTTGAACATCCCCTTCC | TTCCCCTATGTGGCCCAATGCTAAG |
| CYFIP2u1_F1 | ATTTCCTGCCCTTTGTGTGACAGCC | TGCTCCTCTCTCATCAAAGCCCTCC |
| CYFIP2u2_F1 | CCAGGGCCATTCTCACATTTCAACA | TGCCTTTAAGCTTCCCATTGGCTCA |
| CYFIP2u32d2_F1 | TGCTTTCAGGCCATCTCAGCTGCTT | TTGAAAGCATGTTCTGCCATCTGGG |
| CYFIP2u32d3_F1 | ATGAAAGGCCCCTTAGGTCAGATCC | CGTGGGAAAGCAAGCAAAGCCATTA |
| CYFIP2u3c3_F1 | GGCCCCTGAAATTCTCCACTGACCT | GGCCCCCTGGTGCTATCTACCATTT |
| DPP10c10_F1 | CCCTTCTGGTGCCATTTTAGGGAAA | GGAACAGCCAGAGACCGAGAATGAC |
| DPP10c11_F1 | CCAAAGTCCCCACGTATGAAGTGAA | CCTGTTTCCCCTTTGTAAAGCTGCAT |
| DPP10c12_F1 | TCCCTGGTGAGGTACTGCTGGGAAT | GGATCACTCTGTCAAACCTAGCAAAGCC |
| DPP10c13_F1 | GCACAGATTGCATGTGCCTTTCTGA | CGGGGACATGAGGACAATGAAATCAA |
| DPP10c14_F1 | CACCCTCACCTGGAGGAGTTTTCTGA | CCATGGCTGGCTTGTTTTGACTAGGA |
| DPP10c15_F1 | GTCAAAACAAGCCAGCCATGGAAAGA | CCTGTCAAGGGAGAGACTGGTGCAA |
| DPP10c16_F1 | GCAAGCACCGATGTAACTCCCTTGT | TGGCATGGCCTCGCTGATGTAAATA |
| DPP10c17_F1 | TAACCACGAAGTGCTTCCATTTGGG | CAGCTTTCCTCATGGTGGGGATTTT |
| DPP10c18c19_F1 | CCTGAACTACAATCCAGGTGTCCTCA | CCATGTGGGATGCAAGAAGTTCAA |
| DPP10c2_F1 | CAAACTGTGATTGTGCCATGCACTGA | ATTCCAACCCATGCTAATGTGCCCT |
| DPP10c20_F1 | CCCTTGGAAGCCATTTGCAAGACAT | GGAGTCAATGTAAGGCAGTTTCAGCA |
| DPP10c21_F1 | GGAAGTGGATTCCAGGGTCTGAAAA | ACCAACTGGCCTCCTGTGAATGTGT |
| DPP10c22c24_F1 | CCCAAGCCTGAGACATTCTGATATGATG | CCCTATCAAACAGCAGCGGCAAGTA |
| DPP10c25_F1 | TGGGCTGCTTACTTCAGAATCTTGG | GCATGAGTTCATCCACATTCTCGGTG |
| DPP10c3_F1 | CCTTTTTGGGCACAATGTACAACAGG | GGAGTCCTATTTGCCAGGCCAGAAA |
| DPP10c4_F1 | TTAGGCGGGCTTCCTGATCTTTCAC | TATTCCAGCCCCAACTTTTCACCTG |
| DPP10c5_F1 | CTGTTTCACCCTGGGGATTTCCTTT | GGCACTGGGCAAACTGTCCCAATTA |
| DPP10c6c7_F1 | AGGCATAAGGCACAGAGAGGGTGAT | AGTGCATACCAATGCCTGCGCTTAC |
| DPP10c8_F1 | GGTGCACTGGAAGCTGTGTTCCACTT | TGTGGGTTTTATTTTGCTCCCTCTGC |
| DPP10c9_F1 | CCTCTACAGCTGCTTGCTCTTCCTGG | ACACCCCCAATTGCTTCTCTCTCCC |
| DPP10cu26d1_F1 | GGCTCAGAAAATGAATGCGAGAGACA | GATGAATTGGGCAGGCATGGAATAC |
| DPP10u1c1_F1 | GGTGGGGAGAGGGAGAGAGAGAGAGA | TGCAGAAGCAGAGGGAAAAACCAGA |
| DPP10u26d2_F1 | GCACCTTCCAAAGTTCAGCCAGTTATCA | GGGGAAAACGGGTTCCAGAAAAATG |
| ECE1c10_F1 | TCAGCCCATATTCAGGCATACGCAG | CTAGGTGTCCCTGAGCCTCTCTGGC |
| ECE1c11_F1 | AGAGGGCTGTCTGTGAGAGGCGTG | GGGTCACACTGTCCTTTTCCCTTGC |
| ECE1c12_F1 | TTACAGAGAAGCAGCCTGGTGTCGG | AGCCACTGACAGTTTGGGGTATGGG |
| ECE1c13_F1 | GGCTCCCCTAGCTTCAAAGACCTGC | GAATGTGTCATCCCGTCCCTGAGTG |
| ECE1c14_F1 | CTCAAAACAGGAAGAGGTCGTGCCC | CCCCGATGAAGACCAATCCTTTCCT |
| ECE1c15_F1 | GCCAGTGGTACCAGAAGGCTGGAGT | GCAGAGGGAGAGAAACGTGGTCGAT |
| ECE1c16_F1 | GGCCCATTTATTGGTCTCCATGCTG | TGGCCTCTTGTCCTTAACCCTTCCA |
| ECE1c17_F1 | GAGTGAGTCAAGCCCACCATGCAAC | TGTGCTTCTGGGAAGTGTGAGCCAT |
| ECE1c18c19_F1 | GTGCCTTCTGTGGAAAGGGAACTGG | TCCTGCCTGCTAGGGGCTTCAGTTT |
| ECE1c2_F1 | TTTCGGCTCTCTGGGCTCTTGAAAC | TGAGACCTCCATACCAGTCCCCTCC |
| ECE1c20u20_F1 | AGAACAACCCAAAACAGACCGCAGC | AGCCTCTTCCCGGTTCTGCTAGCTC |
| ECE1c3_F1 | CCTCTACGGACAGGCATCTGGAAGG | CTTGACATGACGCCTCCCTCTGACC |
| ECE1c4_F1 | GCCCAGTCTAAAGGGGACCAAGGAA | CCACACCCTTATCCACACAGAGCCC |
| ECE1c5_F1 | CTGGGGGAAAAGAATTGGGGTAGGG | TGAGACCTCCTGCATGTGTGTTGCT |
| ECE1c6_F1 | GTGAGGGTGCAATGAAGGCCAGTTC | ACCGAGGGCACACTGTGATTGACTC |
| ECE1c7_F1 | TGTGTGTTTTGACAACCATGCTGCC | ACTATGAGCCCAGCACCCAAGTTGC |
| ECE1c8_F1 | CAATCATGTGCTTGAGGCCTACCCC | ACGTTAGGAGCGTGGTTTGGAAGCA |
| ECE1c9_F1 | GGAGTGTGAACTGGCAGGTCTGTGC | GAGCCCCCTGACTTGTGTTTTCCAG |
| ECE1u1d1_F1 | TGGGAGGACAGACACCCAGACACAT | AAACTGCTGGACTTACCCTGGGCTG |
| ECE1u1d2_F1 | TGTCCCCTGGCTAAAAGCATCCATC | GTTCTGATCCCCAAAGCCACAGCAG |
| ECE1uc1d3_F1 | GTTCCCAGATGGTGTTTGAGGGTCC | ACAGTGACCCTGGCCTCTCCCTGTT |
| EDN1c2_F1 | CATCCCCCACTGACCTGCTCTTTCT | CAGTGGAGCCAGCGCTAATGAATGA |
| EDN1c3c4_F1 | TGCCCAGTGGAATAGGTGTGTCCAT | TGGCATCACTGACTGGGGCTAGTTG |
| EDN1cu5d1_F1 | TTTATCAAAGAGTTGCGGCGGGTG | TGGGAAGAGTTTGGGGAGGCAATAA |
| EDN1u1c1_F1 | TTTCCTTTCGGGCCTGGCCTTATCT | AACATCTGCTTCACCCAGAAACCGC |
| EDN1u5d2_F1 | TGGCACATTTCAGGGAGAAACTCCA | GCAGGAGGCTGCTACCCAAGAGAAG |
| ERGc10u2d1_F | GAGAGCCTCTTTCCCCTGGAACCTG | TGCCATGCTAGGCCAAGCTTATTT |
| ERGc2_F | TCACCATCATGGAAGCACCACTTGA | AATGGGAGGTCAGGGAGAGAAAGGG |
| ERGc3_F | GTCCTTTCTCCCCCAGTCTCTTCCG | CTTGCTTTGCTGTCGATCCGTGAAC |
| ERGc4c5_F | GTGGAAGGCAGCTGGTCAGAAATCC | AGGCAAATTCTGCACGTCTCCTGGT |
| ERGc6_F | AGATTGCCCAGTGGGAAGTGGGATT | CTACTGGTTGCATGCTTCTGCTCTGC |
| ERGc7c8c9_F | TCAGTCCCAGTGGAAGCAGGATGAA | ACCCAACCCATCTTTCCATTAAGGC |
| ERGu1c1_F | GGAGTCCCATCACATCTGCATCCTG | AGTTCTCAGCGTGGATGTCCCACAA |
| ERGu2d2_F | AAATAAGCTTGGCCTAGCATGGCA | TTCTCCAGGCAATGGGTCACTTTGG |
| ERGu2d3_F | CCAAAGTGACCCATTGCCTGGAGAA | GCATTAATCTGGGCTGCTTTGTCA |
| ESR1c10_F1 | CCATCTCCTGCAATACCAATGGGCT | ATCTGGAGCCTGGAATTGGTCACAA |
| ESR1c11_F1 | GGCACTGGCTCATTGTTACATCCCA | TGTAGGAAGCCCACAGATGCCTCAC |
| ESR1c6_F1 | AAGTGGATCTGCTGCATCTCCCAGA | CGCTTTGGCTCTTAGGATCTGCTCA |
| ESR1c7_F1 | GGAAAACAGCCTCCAAAAGGTTTCCC | AGGGCCCCTGGGAGAGATGTACCTA |
| ESR1c8_F1 | CAGGGGGCCAGAGGCTGTAATATACC | CTCTTAAAAGCTGCGCTTCGCATTC |
| ESR1c9_F1 | CCCCAATTCCTATTGGGCATAAGGC | CATCACCCCCAATGCACTCTTTTGT |
| ESR1cu12d1_F1 | GGGGCTCGGGTTGGCTCTAAAGTAG | GGCCCCTGCTCCTTTCAACTACCAT |
| ESR1u1_F1 | CAAGGCCACCCCCTCCTCTATTTTT | GTAGTCGGATTGCCCTCTGCCTCAG |
| ESR1u12d2_F1 | TGTTCCAGTGGGCACTGTACTTGGA | CCTGGGCGAACATTTCAATCATCTG |
| ESR1u12d3_F1 | GGACTGAGAATCTGGGAGGGCAAAA | AGGCATGGGCTATGGCTTGGTTAAA |
| ESR1u12d4_F1 | GCCATCATGCAGCAATTATGAGAGGC | CAGAGGGAGTGAGGTGAGGAGAGGC |
| ESR1u2_F1 | GGATTATGGCCTGTGCGTGAATGAA | CAACGCATGTAGAGTGCCCATCAGA |
| ESR1u3_F1 | CCATTCATCCAGCGCTGTGCAGTAG | CACATACCCCCATGGAGAACAGCAA |
| ESR1u4c5_F1 | CAACGAGGAGGGGGAATCAAACAGA | CGGCGAGAGAACTTGACTCTGAACG |
| FLGc1d10_F2 | GAGTTGTCTCGTGCCTGCTCATGGT | GACAGCTCCAGACACTCAGCCACTG |
| FLGc1d11_F1 | TTCCGTGGCCTGACACTGATTGTG | TGCAGTCTCCCTCTGTGACTTCCCTC |
| FLGc1d2_F4 | GGAAGCAGACTCAGATCGCCTCTCA | AGAGGAAGGCAGGGATCCCACTACG |
| FLGc1d4_F11 | TGAGTGCCTGGAGCTGTCTCCTGAT | GGAAGACAAGGATCCCACCACAAGC |
| FLGc1d5_F7 | AATGGTGTCCTGACCGTCTTGGGAT | GTCTGGGTCTGCGTCCAGAAACCAT |
| FLGc1d6_F7 | CTTGTCTTCCTCCAGTGCTGGTCCC | CCGACAGCTCTAGACACTCGCAGGT |
| FLGc1d7_F2 | GATCTTGCCTGTTCATGGGATGACG | CCAGAAGTGCAAGCAGGCAAACAAG |
| FLGc1d8_F1 | CTTGTCTTGGACCCCGCTGATTCTC | GATCCCACCATGAGCAATCGGTAGA |
| FLGc1d9_F3 | GGAGCCGTCTCCTGATTGTTTGTCC | AGGGTCCAGTGGTAGTCAGGCCACT |
| FLGc2u2_F1 | GGAAGTCACAGAGGGAGACTGCATCA | AGCTGTAAGTGGGCACAAGGCCAAA |
| FLGu3_F1 | CCCTAAACTTCCAGAACCTTTTGCC | CCCTGCATGGGGCCTGCTATAAAA |
| FLGuc1d1_F3 | GGTGGGAAGGAAATGCCATCTTCAA | GCAGTCAGGATCCAGAAGTGCAAGC |
| GSDMBc2c4_F1 | TCTGGGTCCCTTGAGAATATGGGCA | AATAAAACCTTTTGGGCTGGGTGGC |
| GSDMBc5_F1 | AGCAGCAGTCTTGAGCCACATGGAC | GCAGTGAGGGCTCAGATTGCAAGAG |
| GSDMBc6_F1 | AGGCTGACAGAGCCTGGTTGAATCC | CTAGCGGTGCCCTCTTTTCATTTGC |
| GSDMBc7_F1 | AGAGTCTAAGGCTGGCATCGCCTTG | TCAGTGGGAGGTAAGGCCCTGAGAT |
| GSDMBc8_F1 | AAGATGGGCTTCTTCCCCTCCTTCC | GGGGGTCTGCTCCAGAGTGACCTTT |
| GSDMBc9u9_F1 | TGGCTGGCCACCTGTCATCTACCTT | TGGAACCTTTGGCTCTTCCTTGACC |
| GSDMBu10_F1 | GAACCACAGGCCCATCTCAAGTGGT | TATCTAAAGGCTTGGGAGGTGGGGC |
| GSDMBu1c1_F1 | GTCTACCCCTCAGTCCCACACCTGC | GAGGGCTGCAAATTGGTTAGTCCCA |
| GSTM1c2_F1 | AGTGTAAACTGGGGGCTTCCCTGGT | GAGGGACACCCGTCCCAATTAGACA |
| GSTM1c3c5_F3 | TCTTTGTCCCTGAACCCTGGGATGT | GATGCAGCTCACTGGGGACACTCAC |
| GSTM1c6c7_F3 | GCATCCTTGATTCTGCTGGTCTGGA | CCCATAGATCCAGAGGCTCACAGGG |
| GSTM1c8u8_F8 | AAGGAGTGACAGGGCCTGGTGTGAG | TGGATGAATGAATGGGACATCCAGG |
| GSTM1u1c1_F1 | AGTCTTACTGAGTGCAGCCCCAGGC | TTACACTGCACGTACCCGTTGCCTC |
| HAVCR1c2_F1 | CAGGATCCAAGGTATGCGATGGAGA | GGGACATGGGAAGGAGACACCTCAA |
| HAVCR1c3_F1 | TGCCAGGAACCATTCTCAGTGCTTT | TGGCTGGGTAAGTGATTCTGGGAAA |
| HAVCR1c4_F1 | GGCATCTCTGACAAACTGGGTCACCT | GCAGTGCCTGCAGAATTTGGTCATA |
| HAVCR1c5_F1 | CCCAGGTCTTCAGGAAAGAGAACGC | GGGTGCACATTTTCCTCCACAAATG |
| HAVCR1c6_F1 | GGCCTTTGGGCTTCCAAACACATCT | TGGTGTGCTAGGGTACAGTTCCAGCC |
| HAVCR1c7_F1 | GACTTACGGGAACCTCCTCTTTCCC | GTGAGGGCTCATTCTCAGTTTGTCT |
| HAVCR1c8u8_F1 | CTCTACTCCCTTCTTCCCGCCCAGG | CGCTGGTTCTCTGCTTCCAGTTTCC |
| HAVCR1u1c1_F1 | TGCAATGATCAGAAGGATTGAGCCAG | GGGCTCCTGGAGTCTCGAATACCTG |
| HBBc2u3_F1 | GGGAAAGAAAACATCAAGCGTCCCA | GCATAAAAGTCAGGGCAGAGCCATC |
| HBBu1c1_F1 | GCACTGACCTCCCACATTCCCTTTT | CCAAGCTAGGCCCTTTTGCTAATCA |
| HLA-DRB1c2_F1 | CCAATAGTCCCGCAGAGCACACCTT | AGATCTGGGGCTGGGACATTGTGTT |
| HLA-DRB1c3_F1 | TATGGAGAGAGCCAGCTCCCAAAGG | CAGCTTTCTGCCCCATTTTCAAAGC |
| HLA-DRB1c4_F1 | CACGGTCCCCTTCTTAGTGGGTGAG | AGCAGACACACAGCCAAACCAGGAG |
| HLA-DRB1c5_F1 | CTGCTTGCTCCGGACTGAGAGGATT | AGGAAGACGGAGGATGAGCTCCTGG |
| HLA-DRB1c6u6_F1 | CCATAGTAGCTCAGCACCCGCAATG | GGGTGGAGGGGTTCATAGTTCTCCC |
| HLA-DRB1u1c1_F1 | CTTTGGAGCCAAATGGACCAGATGA | TGAGCCCTTGAAAGAGGGGAAAAGA |
| HLA-Gc5c6_F1 | TAGGATGGTCACATCCAGGTGCTGC | AGACCCCACTTGTCACCCCTTCCTT |
| HLA-Gc7u7_F1 | TGGGTCTGCAGTCACACATTTCTGG | GGAGATGTGGCCAGAGGAGGAATTG |
| HLA-Gu1_F1 | GAGGGGCATTGTGACTGCACTGAAC | GCTCGCCCTGGGATTGTAGGTGTAA |
| HLA-Gu2c4_F1 | CCTTCTTCCTGGATACTCACCGGGC | GGCCAGGCTGAGAGGTCTACAGGAG |
| HLA-Gu8_F1 | TGTTGAGGGGAACAGGGGACATAGC | TCCTCTCCTGCAACAAATCAGCACA |
| IFNGc2c3_F1 | GACCCTCGGCAATGAAACCAAAGAA | GGGGCAGTATTTTATAGTGGGGTGGC |
| IFNGc4u4_F1 | TCACAACTGAATGAGTTCCCACCACA | AAATACCAGCAGCCAGAGGAGGTGC |
| IFNGu1c1_F1 | CCACAGCTAAGAAGACTCCCCTCCC | TGCACCTGGATCTCTGAGGTCTTTGA |
| IKZF3c2_F | GGAATGCTCAAACCTGTAGAAATGTACCC | CTGCAACCAGAATAAGCACATTCCTCTC |
| IKZF3c3_F | CCCTTTTGGGTATGTGTGAAGGGG | TGCAAGTTTTCCTTGGAGAGCTGA |
| IKZF3c4_F | GGGAAGAGGCAATGCATGGCTTAAA | TCCACCACTTCATTTCTCACGTGGC |
| IKZF3c5_F | AAATGCAGCTCCCAACTGATCCCAA | TGGGCCAATCAAGAGGAAAGTTGGA |
| IKZF3c6_F | TGACCTGGAGCCAAAACAGTATGCG | CCACTCCCCAACCTCCTCTACCCTC |
| IKZF3c7_F | CGAGCTTTCCCATCTCGGCATAAGA | TGCCCAACCACTTTCCCTTAAAGCC |
| IKZF3c8u2_F | TCTCTGCCTTGGCAGTGTTCCCTTT | GGGGGAGGCTGAGCCATACTTTTTG |
| IKZF3u1c1_F | TGGGTAGTATTGCGTGAGTGGTGGC | CGTTAAGAGAACCGACGCGCTTTGT |
| IL10c2c3_F | AGCCGAATGTAGCTCCGCAGAAAGA | TCTGTGGATGTGAGTGTCCCTGCTG |
| IL10c4_F | GTGTGATGGACATGGAGCTGGAAGC | CAAGACGGTGAGAGGAGAGGAGGGA |
| IL10c5u2_F | GGCCTGGAGGAGATGAAGGTCAATG | GGGGGTAGCTGGCTTCCTTTCTCTG |
| IL10u1c1_F | AAATCCTGTGCCGGGAAACCTTGAT | ATCTGGCCGATTTTGAAGGGAAAGC |
| IL4Rc10_F1 | CCTCCTCAGGAACTCTGTGCTGGGT | CCATTCCAGAAGCCAGTGAAGGTCC |
| IL4Rc11_F1 | ACCCCTTCCTGAGCATTGCCGTACT | AAGGCAGACATGGAGCTAGGGGCTT |
| IL4Rc12d1_F1 | AACCCTGACCAACCTTTGCTTTTGC | AGGCCTTGTAACCAGCCTCTCCTGG |
| IL4Rc4_F1 | AGTCCCCCTCACGCATTGAGTTCCT | AGCTCACTCTTGGGGCAAACCCTTT |
| IL4Rc5_F1 | TACAGGTGACCAGCCTAACCCAGCC | CTGTTGCTATGACCCCACCTCCTCC |
| IL4Rc6_F1 | TCTGGGTGGTGGCTGTGGTTTGTAG | CACAGAAAAACGTCAAAGCATGCCC |
| IL4Rc7_F1 | GGCTGGTGCCCTAACATCTCCCTTT | CCACAGGGGAAGAATGGAGAGTGGA |
| IL4Rc8_F1 | ATTGGCATGGGGAAGGGAACTAGC | AATGCACCAACTTAGGAGCCGGATG |
| IL4Rc9_F1 | CCAGGCTGTACCATGGCTGACCTC | AAGGGGCAGACTAGAGGGGCAAGTC |
| IL4Rcu12d2_F1 | AGCCAGAAACCTGGGAGCAGATCCT | GTGGGGCCAATCACCTTCATACCAT |
| IL4Ru1_F1 | GACCAAGGGGATCCACTGCAGAGTT | CCCACCCTTTCTCTCTCCGAAGTCC |
| IL4Ru12d3_F1 | TTATCCATGCCTGGGAAATGCCAC | CCTCTGGATGGGTGAATTCCAGGAG |
| IL4Ru2_F1 | GCAGGGGAATTGGCTCTTTTCTTCA | CCCAGGGGGTTCACAGCTCATTAGA |
| IL4Ru3c3_F1 | GGCAGGGGAGGGTTGCATATTGA | CGCCCATCTCAGCTGACAACAACTT |
| INPP4Ac10_F1 | CCTGTCTCCCTTGTCATCCCACCTC | GACAGTCAGTCCTTCCATGTGGCCT |
| INPP4Ac11_F1 | AACCAGTTAGCCCACAGCCTGATGC | GCTGGGAAAGCAGAGGGAGTGACTG |
| INPP4Ac12_F1 | TGCCACAGCATCCTCTGAATTCCAT | AATGGGCAGATGTGGGGTACTCAGG |
| INPP4Ac13_F1 | CAGGCCTGAGCCCAGAGTAATGGAG | CAAGGACCACAAACCCTCTCTGGCT |
| INPP4Ac14_F1 | GCACAGGGAGTGACGCTGCTCTAAG | GTGAGTACGACAACACCCAGCTGCC |
| INPP4Ac15_F1 | AATTGTGGCTGGGGTTGGAATGATG | TTCCAGAAACCCAAGGAAACTCCCC |
| INPP4Ac16_F1 | TTTGCACTGTGGAGAAAGGCAGAGG | GATGACAGCCACAGCAGCAGACTCA |
| INPP4Ac17_F1 | ATCAGTGGAGGGCGATTTGGTTTGT | GAGGCAGCATATGGGAAGCATGGAT |
| INPP4Ac18_F1 | TTGTTAGCCGCCTTACTGCAAGCTG | ATTTCCTCAGCCCAACTGGTCCCTC |
| INPP4Ac19_F1 | TCATTGTGATGACCCCTTCGCTTGT | CTTTTCTGAGCTGAGAGGTGCCCGT |
| INPP4Ac20_F1 | CTGACCCTGAACCTCTTCACCCCAC | GAAAGGATGGAAACCACACACGCAA |
| INPP4Ac21_F1 | CTGAGACTGGGGAGAGTAGCAGGGC | GCCAAACTGCCAACCACAGACCTTT |
| INPP4Ac22_F1 | GCCTCACACTGCTCTCCCTCTCTCC | ACGTTTTCTGCATCATCTCCAGGGC |
| INPP4Ac23_F1 | TGCCTTTATGCTCGGAAGAGGGAGA | AAAGAGATGAATGAGGGCCACAGCC |
| INPP4Ac24_F1 | CACCCACTCCCACGAACTCCTTTTC | TTCTGTCATGACGTTCAGCTGTGGG |
| INPP4Ac25_F1 | TTTCTGTGGAGCCTCGCCTCTAAGC | AGGAGCAGGGTACCAGCTCATCTGC |
| INPP4Ac26_F1 | CCAGCCGAGAGAGTGAACATGGAAA | TTGGCAGACCACGGTTTGACACATT |
| INPP4Ac4_F1 | TTGGTTGTGGGGTCAGGGACTTCTT | CAAGCCCATGCACTGGTGGTAGTGT |
| INPP4Ac5_F1 | GGAGGGGGCGTGAAAAGAGATTGTT | TCAGAGAGAGACCACCAGCACCCAC |
| INPP4Ac6_F1 | GGCACAGCTTGTGTAATGGGCTGAT | CAGCTGACTGCTTCCCACAACACCT |
| INPP4Ac7_F1 | GGACTCTCTGGGGCCATGGTAATTG | AAGTGGGTCCTTTCCCCATCTGTCC |
| INPP4Ac8_F1 | GGACCCTGATGACCCAGTACGGCTA | TCCACGAGGGCTGCATTTTTACTGA |
| INPP4Ac9_F1 | GTGAATGACTTTGTGGCTTGGGCAG | ATTAGGGTGCATGGCTGGGACAATC |
| INPP4Acu27d1_F1 | TGCCTTGTTCAGAAACCTGCCATCT | CACCCCTGGCCCAAGTTTGTTACAT |
| INPP4Au1_F1 | CTACTGTCTCAGAGGGCGGACCCAG | CCGTTAGGCAGTGCAATTTCCATGA |
| INPP4Au2_F1 | TGGAGTAGCTCTTGGCCTCCACGTT | AGTCACACGCTTCACATGGCTCAGA |
| INPP4Au27d2_F1 | CACTCCCATGGGGAATTTATGACCA | GCCCAAGAACAATGGTCCAGTCACA |
| INPP4Au27d3_F1 | CAGTGTTCCTTATGATGCCTCCCCA | TGGTGAAGCCAGGACTCCATTTCAG |
| INPP4Au3c3_F1 | GGAGGAAGAAGTGACCAGAAGGGGC | GGAATCATTGGGTACCTGCCAGCAC |
| IRAK3c3_F1 | TCAGTCCAGACAGCACTCTACCACA | GCAAATCCAGCATTTTTCTGCCTGC |
| IRAK3c4_F1 | GGGAGCTTTGGATTTGTGTTGAGGC | CCGTGTGAAGAGACCACAATTGCAT |
| IRAK3c5_F1 | GGCTTTGGTTCCTGGGCCTTTCTCT | GGCAGGCTAGCACTACCTGACCCTC |
| IRAK3c6_F1 | CATGGCGATACACTGCATGAAAAACC | TCACGCCAAGCTCTGTGCTGTAAAA |
| IRAK3c7_F1 | CCCCCACACCAGTGTGTTGTTCATT | GGGATTCAAGGGGTCAGAGGATCAG |
| IRAK3c8_F1 | CCACCTCACCCCACCTTGCTATCTA | GCCAGCTTGCTAGGAATCCCCATTT |
| IRAK3c9c11_F1 | CATTTCACAGCACAGCTCTTGCACA | CCATGGTTCTGTGGGCTCAGTTTTG |
| IRAK3cu12d1_F1 | CCCACCATTTCCTTATTCCCTTCCA | CCCTTGACCAGCCATTTTCTCAGGA |
| IRAK3u12d2_F1 | GGGTTGAAAGGGGATTTCTTCCCAA | CCAACTGCTTACCGAGGGCCTGTAT |
| IRAK3u12d4_F1 | GCCCATGCAGTTACTCCGCACATA | CCCTTTTCATAGGAGCTTGAACTGGCA |
| IRAK3u12d5_F1 | GCCAGAAAAATGGGGTAAGGGTGAA | TTGCAAGTCAAGCCCACAGACCAT |
| IRAK3u12d6_F1 | CAATGAAAAACCAGTGTTCTGGCAGG | GGCACAACCAAGCAAAGAGCCAAGT |
| IRAK3u1c1_F1 | GAGAACGGCGTGTTCCTAGGGCTC | CTCTGCATCCAACCCAGGAAAGGAG |
| ITGAVc10c11_F1 | TGGTCTGCAACTTCCAGTGTTGTCC | TCCCAAAGCCCAACAATCTCAACTG |
| ITGAVc12_F1 | TGCTCATCCTATGCAAGCCTGTGGT | TCTGCATCTCTCACACCCACAGTGA |
| ITGAVc13_F1 | TGGGTGTGAGAGATGCAGAAAGAGGA | CCTTTCCAGGAAGGAGATTTGCTCG |
| ITGAVc14_F1 | TGATGTGTCCTCCTCCATCCCCTA | GGTGACGGGAATGATGCCTTGAATA |
| ITGAVc15_F1 | TGGCAGTATGTATGTTTGGCAGGTG | CCATCGCAAACTCCGCTACATAGCAC |
| ITGAVc16_F1 | TCATCTTCTTTTCCCTCCCCCGCTA | CAGCCCTACCACCTTCATTTTCAA |
| ITGAVc17_F1 | CCTGCATACCTCTGTGAAGTTGCCA | CCCCAGAGTTCATTATGTCACATTCG |
| ITGAVc18_F1 | CAGGAGTTTCAAGTGGGGCAAACTG | CCTTTGCCAGCAACTCTCGCTCATA |
| ITGAVc19_F1 | AGGCTCAGGGATGAATACAGCTGGC | CAGACATTAAGAGGCTCAGATGGTCA |
| ITGAVc2_F1 | TGCCTTCTGCTTCTCTGCTCTCTAGGC | TTGGGAATAGGGAGAGGAGACCTTC |
| ITGAVc20_F1 | GGCCATGGTCTCTTTTGCATTCAGG | GGGTGCAAATCGAATGCTTCTCTCA |
| ITGAVc21_F1 | GAAGCATTCGATTTGCACCCAGTGT | CACCTGGCGAGTTTGGTTTTCTGTC |
| ITGAVc22_F1 | TGTGGCAATTGTACCCAATTCTGGC | GCAAACCGTGTAGGAGCACAACCAA |
| ITGAVc23_F1 | GGTTGTGGCCCTACTCATGCCTTCT | TGTCCAGAAAAGGCTAAGGCCTGACA |
| ITGAVc24_F1 | GTGTGCACCAGCAGTCAGAGATGGA | TGTCCAGAAAAGGCTAAGGCCTGACA |
| ITGAVc25_F1 | GGAACAAGACATGGCAACAACATGC | GTTGGCTTGCTCACGAGGTTTGAGT |
| ITGAVc26_F1 | GCAAGCCAACGAAGAGAGGAACAAC | CGTGATGGAGCCAAGGTCAAACTGA |
| ITGAVc27_F1 | TCACGTTGTTGTTTCTTCAGGGCA | GGAGGAGGCCCAGAAAGAGGACAGT |
| ITGAVc28c29_F1 | TTTCCATGGGGATGTTGAGATCTGG | CCCAATGATAGGCTGACTTCTGATTGC |
| ITGAVc3_F1 | TGCCCTCAGTGAATAGCAAATGGT | TGCCTTTCTGTGCCTATGAGCTCCC |
| ITGAVc30_F1 | GCAATCAGAAGTCAGCCTATCATTGGGA | TCTTGTTCTTCTTGAGGTGGCCGGA |
| ITGAVc4_F1 | CCGAAAGATCTTCTGGGAATAGGCGA | CCCTGTGCCCTTGAGCATACTGAATTT |
| ITGAVc5_F1 | GCTTTGAAAGTTGGCCAGGTGTGGT | CAAACTAGGCTTCCCACCCCTCTTC |
| ITGAVc6_F1 | GCCCGAGTGAATGACCACTCTCAAA | GATGGGGGATGTCTCCCTATTTCAA |
| ITGAVc7_F1 | TGAAGCAGCCAAGATCCACAAAAGTG | TGGCCCCTAGAGTACAGACATGGAGA |
| ITGAVc8_F1 | TGGAGGGCAGGCTGATTTTTGTGTA | GGACAAGGACGTTATCTTCAGCAGGC |
| ITGAVc9_F1 | CCAATTAGCAACTCGGACTGCACAA | TAGCCTGGACTCTCACCAGTGTGCT |
| ITGAVcu31d1_F1 | GGATTGTTGCTACTGGCTGTTTTGG | AGGGGTCCAGGTTCCTGTCAACTGT |
| ITGAVu1c1_F1 | ACTTCCTCCTCCAGCTCCACAGCAG | TCCAGTTCCAAGTGCGGCCACTTAC |
| ITGAVu31d2_F1 | TCCAAGGCTTTACTGCTGATAGTGCT | ACTGATTGGTGGCAGGCAACACTTG |
| ITGAVu31d3_F1 | TTCCTCAAGGCCTGGGGATGATG | CGACTCATTCTTCTCCACCAAGCTCA |
| ITGAVu31d4_F1 | GAGACTTTACTGGCTTAAGAGGGCTGT | CACGTCACAGAAAGGATAAACCCCA |
| ITGB3c10_F1 | CTTGGCAGGGCAGGGAACAACTTTT | TATATGAGGGGGTGTGGGTTCCAGG |
| ITGB3c11_F1 | CACCTTCCTGGGCTGTGTGTTTTCA | CGGCTCTCTCCAGACTCCACACTCA |
| ITGB3c12_F1 | TGGACTGGGATACGCTTAGGCTTGC | TCACGCTTCCATTTTACAGGTGGGA |
| ITGB3c13_F1 | TGATAGGGGTTTGGAGTGGTCCCAT | ACATGGTTCTCCCTCTCCCTCCTGG |
| ITGB3c14_F1 | CCCTGCTCTGTGCTTCTTCCTCACA | AAAACGCCCAGCCCCGTCTCTTAC |
| ITGB3c2_F1 | GAAAGTTGGGAAGGATGAGGCAGGC | CTCCTCACCATGCATCAATGGGAAA |
| ITGB3c3_F1 | GCTGGACTTCTCTTTGGGCTCCTGT | TGTGCTCTATGCCCACCTGCTTCAG |
| ITGB3c4_F1 | GGACCAGGGCTTTCTGGTTTGCTTT | CCTGGCGTCTGGAGGAGGGACTTAC |
| ITGB3c5_F1 | CATGCTGCCTTTTCCATGAAGGTGT | TGGCACTCCAAGTCCTCCAAACTCA |
| ITGB3c6_F1 | GGGAGCACCTTGGGTCTCTGTTCTC | TGCTGGTGACAGTGAAGGCAGTGAT |
| ITGB3c7_F1 | TGAGCCACAGCCCAAGCAAGATAAG | TCCTGGGGAGTGGATTAGGGGAGAC |
| ITGB3c8_F1 | CAGGACTCTCAGTGGGATTTGGGGA | GGGACTCACAAATCTGAGCCCTGCT |
| ITGB3c9_F1 | GCTCTTGCCATTTCCCGTTTCCTTT | CAAAAGGAAGAAAGGGGGTTGGGTG |
| ITGB3cu15d1_F1 | CGGTGCCTTGGGAAAACTTCTGAGAT | GAGATGGTGCTGGCCTAAGGGTGAC |
| ITGB3u15d2_F1 | CCCTTCAGATTTGCCTTATTGGCAGC | CTGCTCTTGCCAAAGCCAGGTCTCT |
| ITGB3u15d3_F1 | ATTAAAAGCCACCCCCAAATGCCC | CCCACAGGCCAAAACTGCTATTTGC |
| ITGB3u1c1_F1 | TCTAGAGAAGCCGGAGGGGAGGAAG | CACGCTCTCACCCAGGAAGCTACAG |
| LRP1Bc10_F | TCAAATCCCTGTCTCCTCTTCTATGGGG | AAAGGGGTAGCTGCTGGCAAATGTT |
| LRP1Bc11_F | CGGTGGCATTATGCAGTGTAAAGAAG | CTGGAGATACTGATATTCCAAGGGCA |
| LRP1Bc12_F | TGTCCTGGGCTTTCATACTTGTGCC | CCCACTAAGACCATTTGTGTGGAA |
| LRP1Bc13_F | TGGAACTGCCAAATCTGAGGAAGTG | AGCCTGTGGGTTAAAAACAGCAGCA |
| LRP1Bc14_F | GGAGGATCCTTTTGACTCCCAATGC | GGAATTCCCAGGGATAATCACTCA |
| LRP1Bc15_F | TGCTCCCAACCCTCACTTGCTTTCT | TTTCCCTCCCTCCTTGCTCTTCACC |
| LRP1Bc16_F | TCTCAAAGATGGCTGTCACCACAATG | CTATGCAAAGGAGGCAATCTATGGTC |
| LRP1Bc17_F | TTAATCTTGCTTCCCCCTCCCTCCC | GGAAACCAGTCTTCTCAACCCCTGG |
| LRP1Bc18_F | GGAATAGGACAATTCGACCCCATGA | TTTGAGAGTGTCCTGTTAGCCGCGT |
| LRP1Bc19_F | TGTTGAGTCCCTTGGTGCATGGAGT | TTAACAACCATTTCGGGAGGGAGGG |
| LRP1Bc2_F | TTTGCGAGGACTTGAGGAAAGGCTG | AGGTAAATCCGAATGGCATGAGAACC |
| LRP1Bc20_F | GGTTCAGATTTCACTGCCTCATGTTG | GGAGGTATTGAGAGGAGAAACCAAAGGGA |
| LRP1Bc21_F | GGGGCAATAATGTTACCTGGTCATTG | AGCCTGAGCCAAAAGGACTCCACAC |
| LRP1Bc22_F | GAGCACTTTAAGACCAGGGCAAGAA | TGATCTCCCGGAAGAGAAGGAAATG |
| LRP1Bc23_F | TGCACCACTGCTAAAAGGCTCAGCA | ATAACAATTGGGAATGTGGGGCAGG |
| LRP1Bc24_F | CAGGAAGCCGCCATCTTGTCTCTTA | CCATGGAAAAGTCCACAGTGGCAAA |
| LRP1Bc25_F | GGGAAGAGGGTCATCTGGGGTTGAT | CTCACATACCTACCCCCACCTCCCC |
| LRP1Bc26c27_F | TATTCCCCTAATTTGGCAGGCA | GAAACCACTTCCCTCATAGAGTATGCGT |
| LRP1Bc28_F | GGATGCATGCCATTTGTCTAAGAGAG | CCCCCTTTCAAGTTGATAATCCTGTACC |
| LRP1Bc29_F | GGTAACCTGCATAACAAAGAGGCGCT | GGGAAGCCAGTCCTTCCTAATATCC |
| LRP1Bc3_F | TCAATGTCTCTGAGAACAAGAGCAGCAC | GCATTGTTCCACAAATGGACTCAC |
| LRP1Bc30_F | CCTTGGCTCATAAGACTGCTGCCTG | TCTTGGCTGTGAAGAAATCAGATGCC |
| LRP1Bc31_F | GGTTATTGCTGGCAAATGGCTGGAC | CAAGGTTCCCTGTCAGTGCCATCAC |
| LRP1Bc32_F | TCCCCTCAAATCCAGTGGTGCTAGG | TTTGCTCCATGGAAGCCATACCTCC |
| LRP1Bc33_F | GACCGGTAATGGAGAGCACACATGA | TCCCCTAGTTCATTCAGATTCTGGGC |
| LRP1Bc34_F | CCAGCATTAATTAGCACACTGGGCCT | CTTTGGTTGCCTAACATCTGCATGG |
| LRP1Bc35_F | CCCTTATCTCCGGCCCTGCATTTAG | GCAACCAAGTGCCAATGGTGGTATG |
| LRP1Bc36c37_F | CTCAAGCCCATTTGGACAATGAGAG | TGAGAATCTGGAATCATGTTGGTGCC |
| LRP1Bc38c40_F | TGGTAGGATGTTGAGTGCATCCTATG | GTGCTGTCCTGGGCAAATACGCTTT |
| LRP1Bc4_F | TCAACCGCTGAGTTGACATCTTTCC | CCAGCTGCCAGAATAACCAATGCAA |
| LRP1Bc41_F | GCTCAGCATATTTAGGAGGTTAGGCACA | ATTGCTTCTTTGACATTTGGCGGGG |
| LRP1Bc42_F | CGATGTGTTTGCTTTTCCTGGACATC | GGCCTTTTCCAGCATGGTATTGTCTC |
| LRP1Bc43_F | GGTTCTAGGTTTCATTTAAGAACCTGGGG | GCGCCCTAGCATCTCACCATTCTTC |
| LRP1Bc44c45_F | TGCGGTGAACTTCCTGAAGAAAGAACC | CCCAGCACTTTCTAGTGCACAACAGG |
| LRP1Bc46_F | AAGACCAGACGGTGGGGAAAAGTCC | CCACACCGAGACAAGCAAACCAAAA |
| LRP1Bc47_F | TGGTAGCTTCTGCCCCAAATCAGAGA | CCGAATCCAGGAAGGTGGAACTGAA |
| LRP1Bc48c49_F | GCAAGGAGTCATTCTGTCCCATCTTTG | GCCTCAAAATCTCCAAGGCAAAGCC |
| LRP1Bc5_F | TGAGTGTGATTTGCATAGTGTGGCTCAG | ATGCAAGCACTTAAGAGTGGAGCAC |
| LRP1Bc50_F | TGGCCTTAGGCTCACTCAATTTAGAGTCGT | CTCCATTCCCAGCCTCCCCTTCTAC |
| LRP1Bc51_F | GTAGAAGGGGAGGCTGGGAATGGAG | ACAGCTGCCACCAAAGGCTGATGTA |
| LRP1Bc52_F | TAAGCAACATTCTTCTGCTTCCCCC | GCTCAGCCCAAAGGATCCCCTATGT |
| LRP1Bc53_F | TCCCCAAGCTCCTAATATAGGGGAA | TGGCTATACAACTGAACAAGCCAGCCA |
| LRP1Bc54_F | CCGGTGTAGTGTACATTGAGGAGATGACG | TTGACAATTCCCTCAAGCCATAGGTCTC |
| LRP1Bc55_F | GCCCTTGGTTTTCTGAACTTTAGGTG | CATCGATTCTGTCAGCGAATCACCC |
| LRP1Bc56_F | CCCATCAAAGGTCAAATGCTGGGAG | GCGGAATAGAGATGCCCTTAGTCACC |
| LRP1Bc57_F | TTGAGAGGAAGCAAGGGATGCACTTAGA | GGAAGACTAAAGAATCCAACTCTGTCAGG |
| LRP1Bc58_F | ACTCATCAGTTGGCTGTCCAGGGCT | AGCACCCATCCCATTTCCTAGGGTT |
| LRP1Bc59_F | CAACAAATGTGCAGCTCTCCAACAAC | CTCCTGGTATGAAGTGGTGCAGGGA |
| LRP1Bc6_F | TGTGGAAGGAACTGGCTCAGTAATCA | TGCACATACAGCTTTCCAACAGCCA |
| LRP1Bc60_F | GGCAAGATTAATGTGGGAACAGTTGAGC | GTCATTTGTGGCTTCTGCTATATCCGTG |
| LRP1Bc61c62_F | CCTGTGCCCAATCCTGCATTCTCTT | TGGGGACAGTGACATGGAGCAGATT |
| LRP1Bc63_F | TCTCCTGGAAGATCACGTTGTGGCT | CCAACGATTGCTGAAAAAGCAGCAG |
| LRP1Bc64c65_F | TGGAGGCCATCTGAGTTGCTCAAAG | TGGTGCTTCTCTTAAGGATGATGTGG |
| LRP1Bc66_F | GGAGAGGATATCAGGAGTATCTCAAAAGG | TACTCTTCCATCTTATCACTCCAGGCTTC |
| LRP1Bc67_F | CCTCAGCACTTGCCTTCAATTTTTCC | TGTGTGAACTTGCATCAAGGGTTTCC |
| LRP1Bc68_F | GCCCTATGTGGACTATGGGACACCA | AGCTATTCCCTGTGCTGGGGATGTG |
| LRP1Bc69_F | GGAAGCTTTGCCCATGCAAAACAGA | GCCACTGTTTTCTGCATATTGCTGCT |
| LRP1Bc7_F | TCCTTTTCCATCTTCTCCACCCTTTCC | TGATGACCCTGATGCAATTCTTGGG |
| LRP1Bc70c71_F | AAGCCCAGACATTTGAATGCAAGCC | CTGTGTTCGTGTTTGTCATGCCCAG |
| LRP1Bc72_F | TGGATCCTGCTGACTGGCACTGATT | ACTCTAAACCCACCCAAAGAGACCA |
| LRP1Bc73c74_F | CTTGCTCCGGCTCTATGCTGTGTGA | TCCTGGTGGGTATTGCTTCATCCCT |
| LRP1Bc75_F | GCAGCTTATGACCAACCATTATGAGC | GCTCCACAAACAAGCTCTTGACCCC |
| LRP1Bc76_F | GCCCCAGGGAAGTTTGAAATTGAAG | TCCCTTAGAGCAGACCATCAAGAGA |
| LRP1Bc77_F | GCAATCGAACATCTCTTTAGGGCTTCG | AAAGCAGGTGGGGAAAAGGTATGGA |
| LRP1Bc78_F | CCGTGGTGTGTTGTTTTGTTTCCTTG | TCCTTTTTGGCCATTGCCTGTAGGT |
| LRP1Bc79_F | ACCAGGCTAACCTCTCTGACCCGCT | TGCCTGTGAAATCCGACTAATAGCCT |
| LRP1Bc8_F | CAGATGGCTCCATCAAACGTGCTTC | GCAAGAGAGGCATCTCCAATTTTGTGG |
| LRP1Bc80_F | TCTACAGTTTCATGATGGGCACCAG | GAGCCAGGCAGATAGGTGGGCATAA |
| LRP1Bc81_F | CCCCCAGAAAATGCTTTGTGATGGT | CACCCAGGTAGCTGAGCTGGTGACT |
| LRP1Bc82_F | TGTTGAAATGAAGGTCAGATCCCCC | GGGGCTGAGTTGAAACCCATAGCAG |
| LRP1Bc83_F | GAGAAGGCCATGCCAATATGCACAG | GTCACATGGTACATACTCAACCCAGAGT |
| LRP1Bc84_F | CCTTTCATCCTGATGTTAGTCCCCA | AGCTGGCTTCTGGGTGCTGCTTTTA |
| LRP1Bc85_F | TGAAAAGAGGTGTGAAGGAGGCAACA | ACTGAATGGCACAACCCTTTAGCCG |
| LRP1Bc86_F | TTTTAACCAGCCACTCCATCTCTCC | TGCAGATGTGGGTTATATGTGGGCA |
| LRP1Bc87_F | GCTGACTTCTGGGCTTTGTTATTCGC | TGTCACCAGCAGTAAAGGACTAGGGA |
| LRP1Bc88_F | AACTTGTCACATGGACCCCTGACCA | TGCTCCTCAGAGACCCTTGCTGTTTC |
| LRP1Bc89_F | CAGGATTCTAACCATGTAACTTGGGG | TCAGCTTCAGGTGAATGCAGTCCATA |
| LRP1Bc9_F | GGTTCTTCTGCTGTGGCCATTTTCC | CCCCATTCAATCTACCATTAGGAAGG |
| LRP1Bc90u2d1_F | TGCCACGGATAGGCTTGTAATAGCA | GTGAATGGGGTCCATGCGTTCTGTA |
| LRP1Bu1c1_F | CCGATGGCTCGGGAAAGTTCTTACA | CCTCTGCTCCCCATATCCATTCCCT |
| LRP1Bu2d2_F | TACAGAACGCATGGACCCCATTCAC | CATCTAAAGTAGCTGCAGGGCCCCA |
| LTA4H_Ex1_F1 | GGTCCCCCTCAACATCCCTAACACA | GAGGCTACAGAAGAGCAGACGGGGA |
| LTA4H_Ex11&12_F1 | GGGCTTAACTTTCCCTGCTGCCATC | CAGCAGCTACATCCAGAATGAATTGG |
| LTA4H_Ex13&14_F1 | TGTCACCTGGATCCATTCTGCCTGT | TCCTTGTGCAGAAAGGAGGCACTCA |
| LTA4H_Ex15_F1 | TGCTGAGGTGCTTTGGCATTCTTTC | CCCTCCCTGCTTCATGAGTAAAGACA |
| LTA4H_Ex16_F1 | CAACAGTTTCTTCCACTCTTCCTACCCA | TGCATGTGTTCCCCTTCCTACTAGA |
| LTA4H_Ex17_F1 | TGGCTATGCATTTCTCTCCAGCTCC | GGTTGTGGAGAAAGCCTATTTACCACGG |
| LTA4H_Ex18_F1 | CCTGTCATATCCTGCCCCTTCCTCA | TGTCAAGCAACCTACCCTTGACCTC |
| LTA4H_Ex19_F1 | GCCTTATTTGGGTGATGCTGGCTGT | GCAAAGCAAAATCCTAACCATGTTGAA |
| LTA4H_Ex2_F1 | CCCAAAATTTGTGTGACTGTCCTAGGGT | GGGCAAGGAGGGTGCAACTTGTAAA |
| LTA4H_Ex3_F1 | CCCACAGGCTGGCAATTAGGTAGGA | TGCTTCATTCAACCATAGGTGCTG |
| LTA4H_Ex4_F1 | GCTATTTCTTCTTGTTTCTCCCTTTTTCAG | TCAGAATGAGTGCCACAGCCTCTTTG |
| LTA4H_Ex5_F1 | ATGTGCATGGGTGAATGGATAGAAA | TGTAGTACCAGGGCAGGTGAATGCG |
| LTA4H_Ex6_F1 | ATTGCCGGGTAACTCTGTGTGCCTC | TGCCTAAGTTTTGATCTTTCCACAAACCTG |
| LTA4H_Ex7&8_F1 | GCAGCTCACGCCATGTAACCTCATC | GAAACAAAGGTTACACGAACAAGAGAAAAG |
| LTA4H_Ex9&10_F1 | CAGTGGATCACCAGTGCCAGTATGC | CAATTCAGAGTGGTAGGAGGCAGGGA |
| LTAc5u5_F1 | CTGATCCAGACCCCTGATCTCCCAC | TCCCAGACACACACACACAAGCAGA |
| LTAu1c4_F1 | GAACTCTAGGCCTGACCCCACTCCC | CATGTCTGGGAGGTCAGGTGGATGT |
| METc10_F1 | CCAGTTCAATCTGCCTTGTTTCCGT | GCCACATGGTTAGAGGCAAAGATGC |
| METc11_F1 | GGCAGGTGGAGCTCTGTGTTTCTTTG | CATGACGCGGATCCTCTTTAATGGA |
| METc12_F1 | CCTTTAGCCATCCCAATTCCCATGA | TCATGAGAGGAATGCAGGAATCCCA |
| METc13c14_F1 | GGGACCCAAAGTGCTACAACCTGTG | CAACAATGTCACAACCCACTGAGGTA |
| METc15_F1 | ACCTTTGCTGCTACCACTGCTTCCA | TCCCCACGGATATGATTTACAGGGC |
| METc16_F1 | GCCTTTCAGTTTCTGCCAACCTGGAT | GCTCTGAGGGATCATTTCAGGGGAA |
| METc17_F1 | GCAGTCAAACCCTCAGGACAAGATGC | GCCTATTTTGAAGGGATGGCTGGCT |
| METc18_F1 | TGGGGTGATTTCTTTCTGAGCCTCC | ACCCACATCCCCAGGGCTTACACAT |
| METc19_F1 | CTTTCCTCAGCCTGTTGAATTGGCA | CCTAAGGAGCTGGTATGTTGCCCCA |
| METc20_F1 | TGACACAGCGGGAGAGAATTTTGGA | ACCCTTTGAAGGCAGGCATTTCTGT |
| METc2d2_F1 | CCCACAGATAGAAGAGCCCAGCCAG | ACAAAGCACAAAAGAAGCCCTGGA |
| METc3_F1 | TGCACAAGAATGCACAAAGGCACA | CCAGGCTTCATTGTTTGGCTTTCAGT |
| METc4_F1 | TTCAAACACCCACAAGCCCTGCTAA | TGGAAATTCCCTGTGGGAACAGCTT |
| METc5_F1 | CCTCTGGAAGCTCTTTCCACCCCTT | GCCCGAGGGATAAAGCCAAGAGAGA |
| METc6_F1 | GCATCTGCTCTTTGTCCCATCTTTGG | CAAGCACACCCCAGCAAAGCATTTT |
| METc7c8_F1 | TGCTATTCAAAGCAGTCAGCTCACCA | CCACACACACACACAAAACAATCTGC |
| METc9_F1 | AGGCTTCCACTCAGGAAATTCCCAC | CGGAAACAAGGCAGATTGAACTGGG |
| METcu21d1_F1 | CAGAAATGCCTGCCTTCAAAGGGTC | CAATCCATCCACCCTTTCTTTGCTC |
| METu1_F1 | AGAAAGTTTCACCTTGTCGTGGGCG | CTGGATCTGAAAGGGGCTGCAATTT |
| METu21d2_F1 | GCCCCAGAACAGGCCACTCATTTAG | TCACAGTGCAGTGTTTTTGTGGCCT |
| METu21d3_F1 | AGCCACCCTGAGCAGAACTTTGTGA | TGCAGGTAATCAAGCATGACTGGCAA |
| METuc2d1_F1 | CTGCTCTCGCCTTGAACCTGTTTTG | TCGAATGCAATGGATGATCTGGGA |
| MS4A2c2_F1 | TGTCGAGAATGTTGCACAGGGAGTT | CACCTCTCATGAATCCAAGTGGGAA |
| MS4A2c3_F1 | CACTAACGCAGTTTCTCATGTTTGGC | CTCACCCCAGCAATCTCTTTCCTCA |
| MS4A2c4_F1 | CATGCCCTGATGTTGCTGGACG | CAATGCCGGTGTGAGGACACTTTTT |
| MS4A2c5_F2 | GATGCATCCAGCCCTGAAATGAAGA | CCTCACAAGCCTTCTGCACATCCAT |
| MS4A2cu6d1_F1 | TGAGCGAGACTTCTAGGGATGGGAA | CCTTTGTCTCCCCAAAATCAAAGCC |
| MS4A2u1c1_F1 | GGGACAATTCCAGAAGAAGGGCACA | CGTGACTATGACTTCCCCTGCCACT |
| MS4A2u6d2_F2 | GCCTGAATCACCAGCACATTCACTG | GTCGTGATCACTTTCACAATCTAGGG |
| MS4A2u6d3_F2 | CACCTAAGGCATGAATATGCGGCAG | GGAGGACTTCAGAAACTGCTCTTGG |
| MYLKc11_F1 | AAGCCACTGATGAAGTGATGGCAGC | CCTGCAATTCTGGAAAGTGTGGGTG |
| MYLKc12_F1 | AAGTCCTGAGGCCCACGTATCTTGC | AGGAAGTAGGGAATGGGGAGGAGCC |
| MYLKc13_F1 | GCATTTCTCATCCCTTTCGGTCCAA | CTCGCTCACTTTCTCCCTGTCCTCC |
| MYLKc14_F1 | GGGTGTCTCCTGGGATCACATACCC | TAAGAGTCCTCCCTGGATGAGGGGC |
| MYLKc15_F1 | GTGAGTGACCAGAAAGTGGGGGCTC | TTGGCTGAGGTGGGATTTTGACTGA |
| MYLKc16_F1 | GAAATGGGACCCCTGTGTATGGGTG | GGGCAGGTACTACGACCACACCTGG |
| MYLKc17_F1 | CTTCCCCAACCCCCATGCTCATTTA | TCTTACCCAGCCCCTTTTCCTCCTG |
| MYLKc18_F1 | AGCCTCAGCTGCAGGACACAAACAA | CACACAGTGAGAGGGTCCTGCCCTA |
| MYLKc19_F1 | TTGGAGGGAAGGGTTAAGGGAGGCT | GCCTGCTGAGGGACATCCCTGTTTA |
| MYLKc2_F1 | CACAGAACTGACTTCTTGCCCACGG | TAGAGGCGAGGCGAGAACAACGAGT |
| MYLKc20_F2 | CTTGTGATGTTGGACAGGGAGCTGG | TGGCCAGTGTAGCTGGAAGGTTTTG |
| MYLKc21_F1 | TCCCCAAGAGAGAGCTGCAGAGACA | GCATGAATGTTCACCCCACGTTCTG |
| MYLKc22_F1 | CCTTCCTCCAAAGCAGAAGTGCCTG | GAGCCTTCCTGTGCTTCCTCCACAG |
| MYLKc23_F1 | AGTGAGAATTCAGGCAGCGCCAAAG | TGAGCTGACCTGTCCTTTTCCCTCA |
| MYLKc24_F1 | GAATGGTTGTCCCACAGAGAATGCG | AGCTCACGTTCTTTACCTGGCCCTG |
| MYLKc25_F1 | GCTACATTTTGGCAATCGGTGACCC | TATTCTCCCCTCTCCTCACCCACCC |
| MYLKc26_F1 | TCCTCCAAATGGCCAATTCTTCTGC | GAAGTGCCTGGGGAGGGAAATTTTG |
| MYLKc27_F1 | GCGCAGTGAACAAGCAGCTCCTCTA | ACCAAGTTACCCCATTGCCATCCAC |
| MYLKc28_F1 | AGGGTCAGGGCAGACAGAAACCTCA | TGTCTGGGACTGGATTCTCTGCCAA |
| MYLKc29_F1 | TGTACTAGGCTCCCGCCCTTTGGTT | CACCCACCCCTGCTGTTGAATCTTT |
| MYLKc3_F1 | GTTCCCCAGCCCTTTCTTTCTCACC | CCGCTAGGCCCAGTCAGCAATAAGT |
| MYLKc30_F1 | CTGCAGGCCTTGGGGTAACTGAGAG | GCCCTTGGGGGACTTCCTTTCTTTA |
| MYLKc31u31_F1 | CCTCCCCATCCAGCCACACTTCTTA | ACCTGATGCCTGAAGGGAAGACAGG |
| MYLKc4_F1 | GCAACATCCCAGTCCAAAGTGACCC | GACAGGTGGCCTCCCATTTCTGAAC |
| MYLKc5_F1 | GGGCAGGGAGTAGGGAAGCAAAGAC | CCTCTCCTAGCCACACCTGCTGTCC |
| MYLKc6_F1 | AAGTGGTCCACCAGTCCACCAAGTG | TCCGTGATGTGCTGGCAATTTATCC |
| MYLKc7_F1 | CCATGGGAGAGGCAGATGAGCCTTA | GCCAATTTCTCCTACCCCCAGCCTA |
| MYLKc8_F1 | AGAGGTGGCTGGGGTAGGGCAGTAG | TGTGGCTTTTCTCTCCTCCCCCTTT |
| MYLKc9c10_F1 | CCAGAGGCAACTTCCCACTCCACTT | AGCACATCTTGCTCTCAGTGGGCCT |
| MYLKu1d1_F1 | GCCAGTTCTTCACATCTAGGCACCACA | GCTCATGGATGACTCTGGCTTCCTT |
| MYLKu32_F1 | GCCTTCACTCTTACCTGCTTGGGCA | TTGGCTTTCCTTTCCTCCTTCCCAC |
| MYLKu33_F1 | TGAAAGGAAGTGCTTTTTCCTGAGA | GCTGCCTCTGACCTTTTCAGCCTCA |
| MYLKu34_F1 | ATTAGGGGAGTCGGGAGGCACAGAC | ATGTCATGCAAATCAGTGCATGGGG |
| MYLKuc1d2_F1 | TCTGGCTATCTTGGAGGAGATTCCA | GCAACTTGTGTTCTCTCCCTGTGATGC |
| NAT2cu2d2_F1 | CCTGGACCAAATCAGGAGAGAGCAG | GCTCATGTTCCTTCTCTTGTGGCAA |
| NAT2uc2d1_F1 | CAAGTGGGTCATGTACCATGAATACC | GGTCTGCAAGGAACAAAATGATGTGG |
| NOD1c10_F1 | AAAATAGCACCTGGGCCTGCTTCCT | GAGCCTGCCTGAGGGTCCTTATGGT |
| NOD1c11u11_F1 | TTCACTCAGATCAGCAGGGAGAGGC | GGGAGGTGGGGTGAGCTCTTTCTGT |
| NOD1c2_F1 | CGGAACCCAGAAAAGGGGAAGAATG | CCCAAAACACCCTTGCAACTCCCTT |
| NOD1c3_F1 | GCCTGCACTTAGGGAATGGGATCAA | TGAGCTCCCGCTCCTGTGAACTCTA |
| NOD1c4_F1 | ATGTGCAGCCCCTGGGATAATTCCT | GGCTTCCCAGCTGTTCTGATGTTGA |
| NOD1c5_F1 | GACAAGGAAGGGGCAGGAATCTGCT | ACTGGGCACAAACATCTGCCTGTTG |
| NOD1c6_F1 | GTATGAAAAGAACAGCAAGGCCCGC | GTGTACGGTTCCCAAGATGGGGCT |
| NOD1c7_F1 | GCCCTGGGACAAGTGGAAGGCTTTA | ACTCTGGTGCCTGTGTGTTCCCCTT |
| NOD1c8_F1 | CATCCAGGGACCTATGGGAATGTGA | AGGGGCTATTTGGCTGATTGCTGTG |
| NOD1c9d1_F1 | GCACCTTGACCTCTGCCCTGCTAAG | GACTGCACGATGACCCTGACAGATG |
| NOD1c9d2_F1 | TCCTCCTGGGTGAAGACAAAGAGGC | CTCCCTGTTTGATGCTGTTTCTGCC |
| NOD1u12_F1 | CAGAAGGGCAAGAGATCCTCACCAA | ATGAAGGAAGCTGTGCAACACCCCT |
| NOD1u13_F1 | CTACCACGAGGGGCCCGTTTTACTC | ATCTTCAGCCAGGGCTCTGGACATT |
| NOD1u14_F1 | TCCGCTGACTGTGAAGTTCATCCCT | GGCACCACAAATTCCCAAGTGTGTC |
| NOD1u1c1_F1 | TGCTTACCAAGCCAAGTCCCTTCAA | ACCTTGGGAAGGAGTGTGCGAGGAT |
| NOS1c10_F1 | GTCCACAGGGCTTGTCCTTCATCGT | CCAATGCCTGCCTCATCTCCTCTCT |
| NOS1c11_F1 | CCCATTGTCACGAATACCTCCCTGG | CGCTTGGCTGAAAGAGGTCACTGAA |
| NOS1c12_F1 | GGGTGTGGGGAGCATTACTTTTTCC | TCCCCACAGTCCCTTAGCATGAACA |
| NOS1c13_F1 | CTTAGTCTTCCCTGACCCCCGACCC | CCAGGCCAGAGCTCACAGGTTCTCT |
| NOS1c14_F1 | GAACCCCAGGTGCCAAAAGTCAATG | ACCCCAGACCCTCAGTATCCGACCT |
| NOS1c15_F1 | CAGGCTCTGGGAGGGTTTGCTACTG | AAGGTCCCACGCTACCAAAAAGCGT |
| NOS1c16_F1 | CCATGAGAAGCTGGTGGAGCTAGGG | TCTCTCCCCTTTTCTGTTGCCCATT |
| NOS1c17_F1 | GAGGTCAGAGGGCACAGGAGTCTGC | ACAAGTCCCAGGTGGTCCTGAGCAT |
| NOS1c18_F1 | GCCCCCAGTCCCTCTCAAACTCTGT | TACCTCCTGACCCCTGTCCCCTCTC |
| NOS1c19_F1 | ATTTGAAGCTTGACCCTGGTGGTGC | CCCTGTCCCTCTTCAATCTGAAATCCA |
| NOS1c2_F1 | TTCCTGCCCAGTGGGACCTAGAAGA | TGGCATTTGAGAGCTGTTTTCTGGC |
| NOS1c20_F1 | GCTATGTGCTTTTCCCCTGTGGTGA | TCTAGATTCAAGCTGTCCCGCCCAT |
| NOS1c21_F1 | ACTGGGCAAACCCACTCACCCTCT | GTGGGGTACAGTGGCATTACCTGGC |
| NOS1c22_F1 | GACATAAGAGCCCATGTTGGGGCAG | AGTATGTTCCCACAGCGCAAGTCCC |
| NOS1c23_F1 | TCCCTGAGATGAGGTGCATTTCCCT | CTAAAATGGGAAGATTGCGCTGGGG |
| NOS1c24_F1 | TCCCCTTCCCCTCTTCCCTCATTTA | TCCCATGTCCCTTCCTCTTCACTCA |
| NOS1c25_F1 | GCTCCCCGGAATTGACACACACTTG | CAGCCTAACACCTGCCACTCTTGGG |
| NOS1c26_F1 | CCACCCTCTCATCTACACCCTGCTG | CCCTGCCTCATCTTCCACCTTCATT |
| NOS1c27_F1 | TGGTACCAGCTTGGCATCAAGCACT | TCCTGATGTCCCCTTCCTCACCTG |
| NOS1c28u28_F1 | GGCTTCTGGGCTATGAGGCTGAGTC | CAGTTTTCAAGGTGGTGTTGGGCAC |
| NOS1c3_F1 | GGACCTGGGAAGTCAGCCTGTCACT | TCTCACTGCTCTCTGCTCCTTGCAG |
| NOS1c4_F1 | AAGTGTGCATGTGTGTTGGGCTGG | TGACATAAGGTCTTCCTGGTCCCCA |
| NOS1c5_F1 | CTAGGGCTGCTGAGACACAGCTGGA | ACTTCTGACCCCCTCTTCCTTGCCC |
| NOS1c6_F1 | GGTCCACCTACCCCAACTTGTGACG | TGGGTATTTTGCACTGGAGGGAGGA |
| NOS1c7_F1 | GGGAAAATGCAGCTGAGGAGGCTCT | GGCAGCTGGGTTCAAGCTTGTCTCT |
| NOS1c8_F1 | AGAACTGGTGGAAGACCAGCCACG | CCCACCTGTCACCTGCCTTGTACCT |
| NOS1c9_F1 | CAGCTCCCTAGAGCAGGGAAGGGTC | GGCCCTTGTCCTATGATTCCTCTGC |
| NOS1u1d1_F1 | CCCTGATGGAAAAGCATTCCTGCAT | CCACTGGATGACTTTGGATGGGTCA |
| NOS1u29_F1 | CGGCTGAGGAACGACTAGGGTCTTG | GCCCTATCCAGGCTCCCAGAAATTG |
| NOS1uc1d2_F1 | AAGCCCGCTTTGGGGGAATAAAAA | ACTCAGAGGAAACAGCAGCCTTGCC |
| NOS2c10_F1 | CCCATGTGCTGCAGAGAAGCAGGTA | TGCTGCTGACCATTAACCGCAGTCT |
| NOS2c11_F | CCACAGACATAGAGGCCAGTGGCTG | TCCCGTCGTGCCCTACATGTGTAAC |
| NOS2c11_F1 | ATGAATGCACCCTGTCATGTCCCTG | GTCTCTGTGTGTGTCCTCGGGCAG |
| NOS2c12_F | ATGAGAATAAGGGTTAGCCGCCCCC | CTTCAAGGACTGGAGGCAGAGGCTG |
| NOS2c12_F1 | CACACCCAGTTCCATCCCCTGAAC | TGGCCAGGTTTCCAGAAGAAAGGAA |
| NOS2c13_F | CCTGCTGCCATTGTTCCAACCTTCT | AAAACAACAGCCATCCCACTGAGCC |
| NOS2c13_F1 | TAGACCAGCCAGACCTCAAGCCACC | AGAGACAGATGAGCACAGGAGCCGC |
| NOS2c14_F | GAGAGACAGATGAGCACAGGAGCCG | GCACTGACTCCCAAATGTCCTCCGT |
| NOS2c14_F1 | AAAACAACAGCCATCCCACTGAGCC | TCCCCCATTTTCTTCTGGAAGGCTG |
| NOS2c15_F1 | CTTCAAGGACTGGAGGCAGAGGCTG | TGAAGGGTCCTCTACCACTCCAGCA |
| NOS2c15c16_F | AGCTGTGGCCAGGTTTCCAGAAGAA | GTATCTGTGCCACCTTCTCCCCCAG |
| NOS2c16_F1 | AGGAAGGAGAGAAGAGGAAGGCCCC | CCCCCACCCTTATCTCCACTCCCTA |
| NOS2c17_F | ATGTGCCAAGCCCAGACACCTCTTT | TGATCCCTTCTGTCTCCACCACCCT |
| NOS2c17c18_F1 | TGGACCTCTAGAGTGAGCAAGGGGG | AAGGTGCAAAAGTGAAGCCCCCAG |
| NOS2c18c19_F | GACATCTGGGGGCTTCCCCTACCTA | CTTTCACATAAGCTTTGGTGGCCCG |
| NOS2c19c20_F1 | CCGCACTTAGGAAAGGAGAAGGGCT | CCCACCAGGGCTAGGTGGTAAACAG |
| NOS2c2_F1 | CACCTTCAGCCCACACACCCACTAC | GCAGCTGAGGACTGAACCCACTGTT |
| NOS2c20_F | TCACCCAGCACATCTTGGTACAGCA | TCCACATCATCCATCCCCCAATACA |
| NOS2c21_F | TGGGTTGTGACAGTCTTTTCCCCAA | ACAGATGTCCAATAATGGGCGGAGC |
| NOS2c21_F1 | GATGCCTCCCCTAGACCCAAGTGCT | CGGCTGCACTATTGATTCCCAGTGA |
| NOS2c22_F1 | CTCCAGCTCTCTCCCGCTCAAGTCT | ATCTTCCTGCTGTCTTCCTGTCCCC |
| NOS2c22c23_F | TGAGTCCTGAGAGTGCCTGAGTGGG | CTACTATGCGGGAGACGGAAAGGCA |
| NOS2c23_F1 | GACCCTGCAGGAGGCAAGCCTTA | CACCCTCACCACCTGCCTCTCTCTT |
| NOS2c24_F | CCTCTGGTGGCCAACATGGTAACAA | TTTCGAGCTCAGGAGGTGAAGGCTC |
| NOS2c24_F1 | ACAAAGGTGGTTCTCCCTGAAGCCC | GCCTGGGGTCTAGGAGAGACCTGAGA |
| NOS2c25_F | GAAGGGGACTCACTCCTCACCCCTT | CAGCCTTGCTTTGGCCACACAGATA |
| NOS2c25_F1 | CTCTCCAGCCCAGCTCTAACAGGCT | AGAGGAAGAGGGAAGATGGGGATGG |
| NOS2c26u2_F | CAGCCTGGACTTGACACGTTGCTCT | CTCAAGGCCTTCCTCGAAACACTGG |
| NOS2c26u26_F1 | CAAGGAGACAAGCAGGAGGTTCCCC | CCTTCCCTCACCTCTGCTTCCCTTC |
| NOS2c2c3_F | CCGCATCTCATGCTAGCTGTTTCCT | ACCTGTTTGATCCACAAAGCCCTGG |
| NOS2c3_F2 | GGCTCACAGTGGAGCTTGTGTGTGT | CCCTCTCCTGGGAGTGGAGGTAACA |
| NOS2c4_F | ACTTTGCTCTGGATCTGGAGCTGGC | ATGAGGACGTTCAGACACAATGCGG |
| NOS2c4_F2 | GTTTGTGGGGCTGCAGTTCTGCTC | AGCTTCTTGTCCTCTCCACCCCCTC |
| NOS2c5_F3 | GTGACCCCAGTGCCTTCTTTATGGG | CGCGCTTACAGAGGGTGGATCTCAG |
| NOS2c5c6_F | GAGGCATCTTGCCCTTCTGATGTCC | CCCAGCCTTGTCCTGTGTTGAGTTG |
| NOS2c6_F1 | ACAGATGTCCAATAATGGGCGGAGC | CAGTCTTTTCCCCAAAGCGGGACAG |
| NOS2c7_F4 | CCCACCAGCCTTTGACAAGTCTCAC | GCCTCGCTACCTGCTGAGAATCCTG |
| NOS2c7c8_F | CAACTCAACACAGGACAAGGCTGGG | ATGGCTGCAGAATGCAACAGTGACA |
| NOS2c8_F1 | CTCTCAGCAGGAAGGACAGGGATGG | CCTGAGGCTTCATTTTCCTTCCCCA |
| NOS2c9_F1 | CGACAGTGTGGCTGCTAATGCCAG | CAGACATCTGGGGGCTTCCCCTAC |
| NOS2c9c10_F | GGAAGGCCCCAGTTAAATTGTGTCTACCTT | GGACCTCTAGAGTGAGCAAGGGGGC |
| NOS2u1_F | ACCTGGGCATTTTCCAGTGTGTTCC | TCAAATGACCAAACCTCATGTCGGC |
| NOS2u1c1_F1 | CCCTACAAAACACCCACACACGCAT | GCCTGGACTTGACACGTTGCTCTCT |
| NOS2u27_F1 | GCTTCCCCAGAAATGAAGGCAACTC | CTCTCTGGATGGCATGGGGTGAGTA |
| NOS2u2c1_F | GTTGCAAAGCATTGATGAAGGGGCT | CACGAGGCTCTACTGCCTCCCATTC |
| NPPAc2u3_F1 | GTGTCCATCCCATCCCATTTCCATC | CTATAAAAAGAGGCGGCACTGGGCA |
| NPPAu1c1_F1 | AGAAAGCACACCAACGCAGGCATTT | GACAGCCAGGTCACCAAGCCAGATA |
| NPSR1c10u10_F1 | GAAGAACCGAGGCGGGAGGTAGACT | GCGCATGCTTGAGTGTGTGTGTGTT |
| NPSR1c2_F1 | TAGGAGGAAGAAATCCAGCCTGGGG | GCAGGAAGCCATAGGGACTTCACATA |
| NPSR1c3_F1 | CCCCAGGATTTCATTTCTATTGTGGC | CTACCCAGGAGAAAGCGGGCAGTTT |
| NPSR1c4_F1 | TCACCCCTTCTTTGGTTCACCTCTCA | TCCTTAGTGGCTCTGGGCTGTCCAT |
| NPSR1c5_F1 | TGGTTGCCACATACCCAGACATTCC | ACAGCTGGAGAAACAGGAAAGCCCC |
| NPSR1c6_F1 | GCAAGTATAAGGGGGCAGGCATGGT | CGGCTGGGTCCTGATATGATCCTGTA |
| NPSR1c7_F1 | TCCAGAAACCAGCCCAGAACCAACT | GTCACCAGGTCCCAGGAGCAAACTT |
| NPSR1c8_F1 | ATGCTAATGGCTCTCTTCTCCCCCA | TGACCCATGCAAGAGCTCCCCTTAC |
| NPSR1c9_F1 | GCTCTGGTGCTGACCAGTGAGAAGG | GCTACCTTTGTCCCCTCTGCTTGCC |
| NPSR1u1c1_F1 | TGCCAAGGAGAGAGCAGCACGTAGA | TCAGTGTTCAGAGTCGCAGGCAAGA |
| PHF11c10_F1 | GCATCCACCACTAGGCAAGGTCTGA | GGCCAACCTGTTAGACACCTCTGGA |
| PHF11c11_F1 | GCTCTCTTTCCTTTCCCCTGTTTTGGA | GCCACACACAGGCCAAAATATGCTC |
| PHF11c12u12_F1 | TCACCAGAGGCACGAGGATTGTGTA | GGGTCAGACGTGATAGTGTGGGCAT |
| PHF11c2_F1 | AGTTCCACAGGTGGCTCTGACTGCC | CTGGAACGACTGGAGAAGGGGAGAC |
| PHF11c3_F1 | GCCCTTGCGTGTGTGTATAGCTCCA | CCATCACCTACAGAAGTCAAGCTCCG |
| PHF11c4_F1 | CCACTTTTCCATTTCTCACCAGGGA | CCTAAAAGCTCAAACCATGGCCAAC |
| PHF11c5_F1 | GGCATGCAGGTGGGTGTAAGAAACA | TGCATTCCTTGAGGGTCTGTTCATCC |
| PHF11c6_F1 | CCAGGATAAAATGCAGGGCTTCTGG | CCATTCCTTATCCCAATCCCCCAAA |
| PHF11c7_F1 | TCCATCTGCACTCAGTGTGGGTCAC | GTCCCAAAAGTCAAGGCTTTTTCCCA |
| PHF11c8_F1 | CCTCTCCCCAGAGAGTTCCCCATGT | AGGCAACCTCAGGAGCAGCCTTAGC |
| PHF11c9_F1 | TATTTCCCAGCTGCATGACCCACAC | CCAAGTTTGAAACAGGTGGGTTGACA |
| PHF11u1c1_F1 | GATTGTTTCTCCCGCTGTGTTGTGG | AGTGGTGAGCCAAGTTCGTCCAGC |
| PLA2G7c10_F1 | GAGGGCAAGGTCACATTTGAACACC | TCCAAGGCAGCTTGAGAGAAAGGCT |
| PLA2G7c11u11_F1 | GGCGACAGATCAGTTGCTCAAGACC | CCCAAAACAACCACAGCAGCAGAAA |
| PLA2G7c2_F1 | GGGATAGGAGTGAGGCAACCTGCTG | CAGCCTGTGACCCTTGAAACACATC |
| PLA2G7c3_F1 | CCCCCTAGCTCTACCCCCAAAAATC | ATGGTGCGTGTCCACCAATCCTAGC |
| PLA2G7c4_F1 | TGCCCACTGGAGAAGTCTGGCAATA | GGGTGAGGAGAGGCAGAAGTCAGAA |
| PLA2G7c5_F1 | GGGCTGGTTGCAGCCCTTTATCAT | GGAGAGGTGGTTGGTGACCTAAAAGCA |
| PLA2G7c6_F1 | CACCAACCACCTCTCCTTTCACTGC | TGAGGAAGGGAAGGAGCATGCATAA |
| PLA2G7c7_F1 | GGGAGGCAATGTTAGCCATGTTGAA | TGTAATGGCCTCCATGTTCCCAGTT |
| PLA2G7c8_F1 | GGCCTGCATGACATTCCAAACTCTG | CAGTTCAGCTCTTTGGTTGGTGGGT |
| PLA2G7c9_F1 | TACTCTCGGCCACTCCCATCCAACT | TGAAGCCAGGCTGCTTGTATTTTCC |
| PLA2G7u12_F1 | AAGGATCCTAGCGCTGTTTCCGAGG | CGGTACCTCCTCCAGCATCACCAG |
| PLA2G7u1c1_F1 | CAGGTTTTCACTGGGATTTTGCCTG | GGCAGCTGATACAGAGCACATCGTC |
| PLAUc10_F1 | AAACGCCTCCCTGTTTTCTCCACCT | TCGTGCCCCCAGTCTGAAGTGACTA |
| PLAUc11u11_F1 | CCCCTCCTCACTTCTCTAGGGCTCA | CCTTCCCATACAAGCACCAGGGAGA |
| PLAUc4_F1 | CCCTCGCTTACCCCACCTTTGTTCT | CTCCCTGTCCCCAAATTTCTCTCCC |
| PLAUc5c7_F1 | CCCTGTCCAGTCTTCTGGCAGGTCT | GAGTCGAAGAGGAGAAACCCAGGGC |
| PLAUc8_F1 | AGGGAGGGAAGGAAGAAGTGGCAGA | GGTCCCACTCTCCCCAAGCCATTAT |
| PLAUc9_F1 | GCAGACCTCCCTGGATGACTGACCT | TTGATCACGCTGGGCTCATTTTCAG |
| PLAUu1c3_F1 | CAAATCTTTGTGAGCGTTGCGGAAG | GCAGCCAACTGTTGTAGGGGTGGAT |
| PTGDRcu2d1_F1 | CCATTGCTGCCATTAACCTTTCCACA | TAAGTCCTGGGTGGGCTTTTTGCTG |
| PTGDRu1c1_F1 | GAGCTTTTTCTGTGGCGCAGCTTCT | GAACTCAGCAGCAAACCTTCCTGGC |
| PTGDRu2d2_F1 | AGGAATCAAAAGTTGGGGGTGGGT | CCACACTAGGAAACTGCCAGACAATGA |
| RCBTB1c10_F1 | TGGAGGCAGTCGAAAACCTCAGAAG | TCAAAGATGGAGAAAAGGCGGGGTA |
| RCBTB1c11u11_F1 | CCCCGCCTTTTCTCCATCTTTGAAC | GGCTGGGGGAAAGTGAGTGATCCAT |
| RCBTB1c2_F1 | GGGGAGACGAGCACAAAGGGAGTTA | TGTGGAAGTGGAGGAGGCAGACAGT |
| RCBTB1c3_F1 | TCGTCCATCCCAATGCTACCACAGT | CCTGGTGTGTTCAGGCCCAGATAGT |
| RCBTB1c4_F1 | CAATGGCAACCTTTGTGGCAAACA | GAACCACTCGTTTTGAGAGGGGGTT |
| RCBTB1c5_F1 | GTGGAGGTGGCCCTGCACTTTCTTA | TGGTGTGCTGTGGGCTTCTGTGAT |
| RCBTB1c6_F1 | CACATCGCACACACAGTCCCAAGAC | CCTGCCCTTCCTGTTTTCCTGCTAA |
| RCBTB1c7_F1 | AGCAAGGAAGGTAGATGGGAAGCCA | TGTTTGGAGATCAGTTGCAGGAGCA |
| RCBTB1c8_F1 | GGGAAATGGGAGTGGAGACACCTCA | CGCATTTCCTATTGATGCCACATTCG |
| RCBTB1c9_F1 | GAGCATGGGACTCAGCTGCCTTAAA | GGGGCAATGGGAGAGAAATGAAGGT |
| RCBTB1u12_F1 | GCAAACGAACCAAGTGGCAATAGCA | TTTGACCCACCAATTCCAGCCTTG |
| RCBTB1u13_F1 | GGGAGGTACAGGAGTAACGGGGCAC | AAAAGCCTCTAAGTCCCGGCAGCAG |
| RCBTB1u1d1_F1 | GCCTTTCCCTTCAAGTACCCCCAAA | GACCTGGGGATTGTTAAGTGCTGCC |
| RCBTB1uc1d2_F1 | CATCCCAAAGTCACCTTCCATAACCA | GGGGAGGGTGAGAGATTTGGGGTAA |
| RUNX1c3_F | CCTGCCTAGCAGAGAGAAGGGAAGGA | GCTGTGAGGGAAGCCAAGCTCTGTT |
| RUNX1c4_F | GGCCCCTGAACGTGTATGTTGGTCT | GGACACCCTGATGTTTTCAGCCCTC |
| RUNX1c5_F | CCCTGATGTCTGCATTTGTCCTTTGA | TAAAATGCTAACCAGGCCCAACCCC |
| RUNX1c6_F | ATTGGGAAAGATGGGAGCAGGGAAG | ATGCAACTTTTTGGCTTTACGGGGG |
| RUNX1c7_F | CTGGGTTTGCAGTAAGGAACAGCCG | GAAACCCCAGTTGGTCTGGGAAGGT |
| RUNX1c8_F | TTAGACCTCATTGCCGGTTTCCCAG | AGCCACTTCTGCCTTCACATCCACA |
| RUNX1c9u2d1_F | CAACCTCCTACTCACTTCCGCTCCG | GATGTTGTTGAGTGAGCAGCACAGGA |
| RUNX1u1c1c2_F | CCCGGAGCTGATGCCTAGCATTTTA | CTCTGCACCGAGGTGAAACAAGCTG |
| RUNX1u2d2_F | TCCTGTGCTGCTCACTCAACAACATC | AAAGACCCAAACATGTCATTCCCCC |
| RUNX1u2d3_F | GGGGGAATGACATGTTTGGGTCTTT | TGCAGTGGGCCTTACTCAGTTTCCC |
| RUNX3c2_F1 | AAGGGGGTGCAGTGACTGATCTTCG | GAAAACCTCTGTGAAGAGGCTGGCG |
| RUNX3c3_F1 | AGAGGGTTTAGGGGTTTGCCAGCAG | ACCTGCCTCTATTCCCCACTCTCCC |
| RUNX3c4_F1 | CCCTCTTTCAACCTCCTTCCCTGCT | TCCTTTCCCTGGTCACCCTCTTCCT |
| RUNX3c5_F1 | CGTTAAAAGTCATTCCTGCAGGGCG | TGGGACCCGGACGTTCTAAGCAAAT |
| RUNX3c6u6_F1 | CACAGAGACCACCAACCCCTCTTCC | AGCCTGGGCTCTGTCAAGGGTAAGC |
| RUNX3u1d1_F1 | ATAGCCCTAGAAACCGGGCACTGGA | ACTGCGTTCCGAGGTGAAGTCTTGC |
| RUNX3u1d2_F1 | CCACAGAGGCCGAGGCAGAAAGTTA | GTCCAGCCCCCTCTGCATTTACACA |
| RUNX3uc1d3_F1 | GGGACTGGCTTGGATGTGTTCTCAA | GCTGGAACTGACCTCTTCCCTGCTG |
| SCGB1A1e2_F1 | AGAGGGCTAGCCAGCTTGGAAAGGG | CCAGTGTGACGTGAAAGGTGCTGGT |
| SCGB1A1e3u3_F1 | TCTGTGGCTCGTCATCTCTGCCTAA | AACAAGGAGACCAAAACCACGCAGG |
| SCGB1A1u1e1_F1 | GGGCATGTCTCATACTAGCCCACCA | AAGTCCAGGAGGGAGGGAAGGGTCT |
| SELPc10_F1 | TGCTGATGCCTCTAACGAGCTGAGA | CGGCCCTTCCATTACCTGGCTTTAG |
| SELPc11_F1 | CCATGCCATGATTCCTTCTTGGACA | CCACCATTGCTCTCTGTGTGGCTTT |
| SELPc12_F1 | TTTTGGCAATTCAGGCCAGCTTCTC | TGAAGGCTTCCAAGGTGATGGACAA |
| SELPc13_F1 | GCTCATTGTGTCCTCTTCTCCCCCA | GGGAAAGGGAAAGGAAGCAAAACCA |
| SELPc14_F1 | TGGTTTTGCTTCCTTTCCCTTTCCC | GGGGATGAGTCTGTGGTCAAAACGA |
| SELPc15_F1 | GCCAACCAGAGAAGGCAGGGTTTTT | GGTGAGGGGTACTAACCTGGCACTCA |
| SELPc16_F1 | CCATGCCTCAAACCAACTGGCTACA | GCAAGAAGGAAGCAGAGGGCTTGAAC |
| SELPc17u17_F1 | TCCATGGCTATCGCTGTTCCTCACT | CTTCATGGGAAGGGCTGTGGTGAGT |
| SELPc3_F1 | TGGGGGCATACTGTCCCTTTTTGAA | GGGCAAGTGACACAAGATGCCCTC |
| SELPc4_F1 | TGGTTTTTGCCTCCCCACCCTAAAT | CATTCCCAGGACCTTTGGTGATCCT |
| SELPc5_F1 | CGGGGATGGAAAGCAAGACCACTAT | CCCTTATGAAATCCACACCAATCCCA |
| SELPc6_F1 | TCAGAACTGCCTGCTCCTTTTCACC | TTGCAGGAGCCTCCCTTGTTATGAA |
| SELPc7_F1 | TGGAGACCATTCCGTTCACTCAACA | CTTACCATCCTCCCACTGCCCTTTG |
| SELPc8_F1 | CCATAAGAAAGGACAGACCCACAGCC | TTTCTCCTCTGCCATGCCCATGAT |
| SELPc9_F1 | GAGGCATTTGCATTTGGGATCCTTG | CCCAATTCAGGAATCCCTGTTCTTGAC |
| SELPu1_F1 | TCTCTTGCAGTGTAGGGTGTGCGAT | CCTGGTCTCCTCATTCAGCCTCCAT |
| SELPu2c2_F1 | CTGCCAACACAATTTCCTTCATGCC | GCCCCATACCCACTCAATATCGGAA |
| SETDB2c10_F1 | AAATGCCAGGTGGTGTCCAGGAAGT | TCCAAAACTGGAACACTGAGGATGG |
| SETDB2c11_F1 | GCCAGGTGGTGTCCAGGAAGTATCA | TTACATCTCAGAAGGGCCCCAGCA |
| SETDB2c12_F1 | AAGGGGATGACACTTGTTGACTCCA | AGTTTTCCAGCCCACCCCTCTTGTC |
| SETDB2c13_F1 | TGCCTCCAAAACACCCACTCTGTTA | TAGCCGCCATCTCCACACATTCATC |
| SETDB2c14_F1 | GGGCTTTTCTGAATGGGTTCCCACTAT | CCGAGTGGAACTGGGTGATACCTGA |
| SETDB2c15u15_F1 | TGGTGGATGTTGGGTGAGAGAACAGA | ATTAGATCCTCAGGCGCTGTTGCCC |
| SETDB2c3_F1 | CCCACAACCACTCTCCAGTGAAGTC | CAACCAACCAGGGTTGACAACAAACA |
| SETDB2c4_F1 | CCATCAGGACATACGGTTTTGAAGCA | CCTAGGCTGCTTAGTTGGATGTGCATT |
| SETDB2c5_F1 | CCTTTCACAGTATCCTTCAGTAACACTCGG | TGTGTTCTTCCAGAAGTGCCCCT |
| SETDB2c6_F1 | GGATCCATATGGGCAGACTCTAGTCAA | CCGCACATCTCCTTTATGCCAAAGA |
| SETDB2c7_F1 | CTCAGCATGCCTGAAGAAGTAACCT | CCTACTGCTAGTGACAGAGTTCATTTTCCC |
| SETDB2c8_F1 | TCTTTCTCCATCCATTGCCTGCCTT | CACACCCTGCAACAAATCCTCAAGGT |
| SETDB2c9_F1 | CTTTGGGCAGCCATTTCTCCGTTTA | TCAACAGCCCTGGCTAGGAAAATGG |
| SETDB2u1_F1 | GCGGGACTGTTGTGGTTGAGATGAA | GGCTGTGGCTGATAGCGAGTCAGAA |
| SETDB2u2c2_F1 | TGATAGGATTGGGTGGGCCTTCTCT | TCCTCGCAATGCAAAACCTTGACA |
| SPINK5c10_F1 | GGGTCTTTTTGGGGATTTGAGGTGT | GCATCGCTGACTCGTGGAACCATAG |
| SPINK5c11_F1 | TCCTCTTGGATGGCCTTTTTCCACA | CATGTTTGTCAGGACATTCCAGTGC |
| SPINK5c12_F1 | TCCCAGATCCACTATTGCCGTCTTTC | TCTGCAGGGCAAAATCTCACCCTTT |
| SPINK5c13_F1 | TGACACCTTCCCTGGTTTCCTTTGA | ATGTTCCCAGCTGCAGGGCTACTCA |
| SPINK5c14_F1 | GCACAGGGTTAGGCACATCACATTCA | GTCCGTTTTTCCTGGATTTGCGGTA |
| SPINK5c15_F1 | TCATGTTCAGGGAACACGTGCATTC | CCTCAGACGCTATGGGGCTACTGCT |
| SPINK5c16_F1 | CCACCAAGCTCAATTTGCAACAC | CTGGACACTTCTTATCAGGACCCTTT |
| SPINK5c17_F1 | GGGACAATTCTGATTGATGACGGAAGC | AATTCAGTGGGAGAGATGGGGTGGA |
| SPINK5c18_F1 | TCACCTGAGGGCCACAGACTGATAA | TGGGGATCCAAACACATTTTCCCAG |
| SPINK5c19_F1 | AACCTGCTTCTGCTTCATTTGGCA | CCCACACTCAGTAGCAGCAGTGGCT |
| SPINK5c2_F1 | GTCCAGTGCCCTTCTTTTATTTGCCA | TGCCTCTTGCAGACATCCATCATCA |
| SPINK5c20_F1 | TGGTTTGCTATGGCATGTGCGTCTT | CCCTAGTTCCCTCACAACTGGGGTT |
| SPINK5c21_F1 | GCAGGTTTAGTCTGGATTTTGGGGC | CCCCAATTCCACCATCCCACAGTAG |
| SPINK5c22_F1 | GGGGCCTCTTGAACATTAGGTATTGGC | TCCAAATGAACCCCAGAGCAGTGAA |
| SPINK5c23_F1 | CCAGGGGCTCTTCGTTCTTCTCTGT | GCATGGAAGGAGCCATAGCAAATGA |
| SPINK5c24_F1 | TCTTGTCATTTGCACGGGACACACT | CCAAGAGTCAGGCAGCCATGTAACC |
| SPINK5c25c26_F1 | TGGCTGCCTGACTCTTGGAAAGAAA | TCACTCCTGGGGAACTGAAGAGCAT |
| SPINK5c27_F1 | GGGGCTCTGCCTCATAGAAAATGGT | CCTTTGCATTATTTGAGGGCTTGC |
| SPINK5c28_F1 | CCAGAACCTCTGGGGTAGGAAGAGG | ACCCCCAAACCCCCAAAAATCTTGT |
| SPINK5c29c30_F1 | GGAAGGATGGATGAACGGGAGAAAA | GCTAGCTACAGGGGGAGCAGAATCC |
| SPINK5c3_F1 | CCTTGCCTCAGTCCTTCAGCAACAT | GGCCCTCTGGAATGCTTTCAGAATC |
| SPINK5c31_F1 | GTGCACGGTGCTGATGGAAAGTTCT | GCACTGAATCAGCAAACCTCAGACCC |
| SPINK5c32_F1 | CCCACCCCTCTTCTTGAATGCCATA | CTGGAAGATATGATGGGGCAACTTGA |
| SPINK5c33_F1 | CCCAAGTGTCCTGCATGTTGGTCC | TGGCAGTGGAATGGAGCAAAGAGAA |
| SPINK5c34u34_F1 | GCCCACATTTCTGCAATATCTCTGGGT | ATGGGAACCCATATCTCCCCACCTC |
| SPINK5c4_F1 | ACATGCCAGGCTAGGCTGAGGAGAG | CCCTCCCTCCTAAGTTGTTGGCTTCA |
| SPINK5c5_F1 | CTTTCTCCTGCCGTACCTAAAAGATG | GAGAACACGTTTTAGGGAGAAAGCCT |
| SPINK5c6_F1 | TGGCAGCTGTTTTCGGGAGTAAAGG | GGCTCCCACCTAAGAAGGAAGAGGA |
| SPINK5c7_F1 | GCCCATAGCAATGTCAGAGGGACTG | TCAAGTCCACCTGGGGATGATGCTA |
| SPINK5c8_F1 | CAGGCATTGAAGACGGAAATGCTTGT | GTGGAAACTGCACATTTTGGGCCTT |
| SPINK5c9_F1 | TCCCCTGACCCCTATTGATCTGAAA | TCAATCTCCACTCCCACCCTTTGAA |
| SPINK5u1c1_F1 | TGGTTTTATGTGGGTGTCACCAGTGC | GCTTTGAGATGTGTGGCAAGAGGCT |
| TBX21c2_F1 | GCTGGGGGCTTTCTCTCTTCTCTCC | TGGAGTGGGAGGGGGTTAAAGGTCT |
| TBX21c3_F1 | CCCTACATCACCAGGGTTCTGTCCC | AGCAGGGCGAGGCTACCTTTTGAAG |
| TBX21c4c5_F1 | GAAATCCTTCCTCCCCACCCCTACA | TGTGTCTCAGGAGGGACCAAAGCTG |
| TBX21cu6d1_F1 | CTGCTTGTGACCCGTTTTCTTGCCT | AGCCCCACTTCCCCAACCAACTACT |
| TBX21u1c1_F1 | CAGCAAAGCGCTGCGACTCTAGTGA | GGACACGGAGTCAGAAGCATTGTCG |
| TBX21u6d2_F1 | CTGGGGCCCCTTCTCCTTTTGATAA | TACAACCCCAACTGAGTCCCCATCC |
| TBXA2Rc1d2_F1 | CCCAGCAAGTAGGTCAAATTCAGGGTC | AGGCTGACAGCTCTCCCCTTTGCAG |
| TBXA2Rc2u2_F1 | CAGTCAGGAGGGTAGCAGGACCCC | AAGCAAATCTTCTCTCGCCTCCCAG |
| TBXA2Ru3_F1 | ATGTGGCCCCACAGAGACCAGAGAG | GACAGAGACTGGACGGAGAGAGCGA |
| TBXA2Ruc1d1_F1 | TCACTCCACGCTTTTGCATCTCTCTC | AAGGGCATGCAGACATTGGAAGAGG |
| TEKc10_F1 | CCTCAAAGCCGAAGGACTAATCTGCC | CCAGCTGGCATCTATCCTGTCCAAA |
| TEKc11_F1 | TCGCAATAACAACAACCCCGGCTTA | CCCACCCAGCTCATGAATAAATGCC |
| TEKc12_F1 | TTTAGCGCTGTCATTGACTCCACCC | CAGATGAGGCTGGAGCAGCATGAAT |
| TEKc13_F1 | GCGTGGAGTCCAGTAGCCACTACCA | TGCCTATAGGGCTGCACGGTAATCC |
| TEKc14_F1 | TGCTTCACCTCTGTCTTCCTGCACA | GGTTCTGCCTGTACTTGGACTTGCCC |
| TEKc15_F1 | GCCAACCAGAAGACATTATGCCCCT | CACACCCTCACGCAATGCTGACTCT |
| TEKc16_F1 | CAGGACGGGACAGCTGATTCTGAAA | TGGCAGGAAAGACAGGAAAAGAGCA |
| TEKc17_F1 | GTCGATGCTCTCTTCCTTCCCTCCA | CCAAATGACACACCACATCTTGGCA |
| TEKc18_F1 | CAAAGTTTTCAGCCCTGGGGCTTTC | ATGCGATCAGTGTCTGTGTCTGGCA |
| TEKc19_F1 | CGCTGCTGGACCCCGAAAGATAAAT | CCTGCCACAAGTGATTCTGCACTCC |
| TEKc2_F1 | GGCTTTGTGAGCACCAGTTTACCCA | GTGTGTGTGTTCCTCAGTGGGCCTC |
| TEKc20_F1 | GAAAGGGTGGGTCTCCCTGGCTTTT | TGCAGCAAGGGAGTGCTAGAGGCTT |
| TEKc21_F1 | CCATGTCTTCCTCCTGGCCCCTTAT | GCTAGAGTCAGCTGGCCCCAGAAAC |
| TEKc22_F1 | CCGTGCCTAGGACTGAAGCACAGTT | ACCACCCTGGGCACATCAGGTATTC |
| TEKc23u23_F1 | CTGGCAGAGGTGGAATCAAAGCAGC | TGAGCTTCAGATTCCGTAACTGGTGGA |
| TEKc3_F1 | CAAGTGCCAGCCCTCATTTTCTGAG | TCAAACATCTGCCCACAAGACCACA |
| TEKc4_F1 | TGGAGAAACCAGACAAGGAAGCAGG | CACAAATGGTGGCCTCTCCATCACT |
| TEKc5_F1 | TGGGAGAAGCAAGTGACATCCCCTT | CGTCCATTCCCATCTGTGTCGAGAT |
| TEKc6_F1 | TGTCTGCTCTGTGGGGCAGTTTCAT | TGTCCAAGGGTGTGATTGGTGCATA |
| TEKc7_F1 | GCAGTCTTAGTTTCCTCTCTTCCCCTGG | TGAGGATCTTAGAATGCCGGCACAA |
| TEKc8_F1 | GCTCTGAGGGCACCTCTATCGCCTA | TGCAGAGCAGTACTGGCCAACAGAA |
| TEKc9_F1 | GGGGTCAATGTTATGGACCTTTGCG | GGAAGTCACAACGGCAATGCTAACC |
| TEKu1c1_F1 | TCTGAGAAGGATTGGTCATCATCGCA | TTGGGGAGAGCACCCAGAAATCACT |
| TGFBR2c2_F1 | TGGGTGAAAGATCACCAGAGGGAAA | CCTGATGAGTACACTGCCATCGCCT |
| TGFBR2c3_F1 | CATCTTCAGGAATTCATTGGCAGGC | GGGTGCTGAGGAGGTGTCGGTTAAA |
| TGFBR2c4_F1 | CCCCTCGCTTCCAATGAATCTCTTC | ATTGGCACAGATCTCAGGTCCCACA |
| TGFBR2c5_F1 | TCAGGCATGAACCCACTTCCTGACA | GCCAGGCTCAAGGTAAAGGGGATCT |
| TGFBR2c6_F1 | GATGGGCCTCACTGTCTGTTTTTGC | CCCCTGGAATAATGCTCGAAGCAAC |
| TGFBR2c7_F1 | TCAGTGACCCTGTGTTTGCTGGCTT | CAGGGCCATAGAACACAATGGGACC |
| TGFBR2cu8d1_F1 | GCCCTTTGGATCTCTTTCCCGCTAC | GCAACCCATGAAGGTAAAAAGTGGGG |
| TGFBR2u1c1_F1 | GGCAGCTACGAGAGAGCTAGGGGCT | GCCCCTTGCAACTGAACTTTCCTTC |
| TGFBR2u8d2_F1 | CGGGGTTCCTGTGTGCCCTTATTTC | TTGAGAAGGCTGGCATGATGGAGAG |
| TGFBR2u8d3_F1 | CTTTCCACTGCCTACCTGGACCCC | CTTCTGGGCATTCAGAGGGACATCA |
| TGFBR3c10_F1 | TCACTGGAAAAGCTTCATTTGGGGG | CCCAAGGAAAACAGGGGTCAAAAGA |
| TGFBR3c11_F1 | CTGAGGTCCTTGTGGGATGCTCAAT | GGCCTGGGATCAACTCCAGCTTTTT |
| TGFBR3c12_F1 | GAAGCATCAATGGCTCCCTTCCCTC | TCCCTGTGTCTGCCATTTGCTCAGT |
| TGFBR3c13_F1 | TCCTTGCCCTAACCACCTGGTTTCA | GCTCCGGTGACTGCACCATGTTAAA |
| TGFBR3c14_F1 | GATGGGCAGGTGTGTCTGCTTTCTG | GCCAGGGCTAAGATTTCTGCCCTCTT |
| TGFBR3c15_F1 | GGCTGAGAGCTGACACCTGCACCTA | GCTTGTGTTTGTGATTCCCTTGGCA |
| TGFBR3c16u16_F1 | CCCAAGCAGCCTATGTCAAGAGTGTCA | TCTTGCTGGCACTAGGGTTGCATGT |
| TGFBR3c2_F1 | GGCAGGAACACAAGCTGTTCACCAA | CCACTGGGAAGTATTTCCGTTCCCCT |
| TGFBR3c3_F1 | GGGAAGGGGGAATGAGAGCAGAAGT | CACACACACAAATTGTCGGACCCAA |
| TGFBR3c4_F1 | TTAACAAGCAGAGCTCAGGCCCCAT | AGGCACTTTTGAGTGGCCCCTGATT |
| TGFBR3c5_F1 | TAACCATCCGAATGGATGAAGGCCC | CGTGTCATCTGAAGGGCTGTCTTGG |
| TGFBR3c6c7_F1 | TGAGCCAATCCCTCATCAGACCTGT | GTGAGGCAGAACCAAACACACATGG |
| TGFBR3c8_F1 | GAGAAAACAGGCCTTCCAATTCCCA | GCCTCCAACCATGTCTTTGGTGGTT |
| TGFBR3c9_F1 | AGATGCAGACTAGGGCCAGATGGGA | GCATCAAACACTGCTGATGTTTTGCC |
| TGFBR3u17_F1 | ACCCTGGCCGGTAAGTAGGCACTG | AAGGACCTCGTTTGAGGGAGCGAGT |
| TGFBR3u1d1_F1 | GGAGCAGACGATCCATTCAGGAGAAA | GCTTTGCCTAACCTTCGGTGGAATG |
| TGFBR3u1d2_F1 | GCAAATTAAGGGTGTAGCCTGCAGAA | GGAACCACAGATTTGTGCCCATTCC |
| TGFBR3uc1d3_F1 | ACTGGAGACCGACAGGATTTGCCAT | GTTGCTTCTGCCCTGGTTGAGAAAA |
| TLR4c2_F1 | TGCCAGACAGTCTCCCCAATGATATG | ATGGTGGAGAGGGGCCTCTTTTTGT |
| TLR4c3_F1 | GGGGAGTTGGGAGACCATGCAGTAA | CCACAAACCAAGCTTTCCAGTCCAA |
| TLR4c4d1_F1 | CCCATCACATCTGTATGAAGAGCTGGA | TGCCATTGAAAGCAACTCTGGTGTG |
| TLR4c4d2_F1 | TCAAAGTGATTTTGGGACAACCAGCC | GGTCTTCTCCACCTTCTGCAGGACA |
| TLR4c4d3_F1 | GCAGTTTCTGAGCAGTCGTGCTGGT | TCTTCCCGTTTAAAAGAGTGCCCCC |
| TLR4c4d4_F1 | CATGCTCACAACCATCCTGGTCATTC | CCTGCTCAAACCAAACACACTCTGA |
| TLR4c4d5_F1 | CATCCAGCCTCCTCAGAAACAGAACA | TGTCAGCCTTGAATATTTCGGCCTTG |
| TLR4u1c1_F1 | GCCCAGGCAGAGGTCAGATGACTAA | TCAGAGGAGCTGACTCCAGCCACAT |
| TLR9c1d2_F1 | AAGGTTGAGCTCTCGCAGCTCCTTG | GTGTCCTTTGCCCACCTGTCTCTGG |
| TLR9c1d3_F1 | TCATCGAGTGAGCGGAAGAAGATGC | GAGTGGGTGGAGGTAGAGCTGGGG |
| TLR9c2u2_F1 | CCAGGGGACTGAGAGCTGTTGTCCTA | AGTGACGTGGTGTGTGCTCTGATGG |
| TLR9uc1d1_F1 | GCCTCCAGTGAGCTCAGAGTTAGCCA | TGTCAGCTGCAACAGCATCAGCTTC |
| TNFc2c3_F1 | AGACACCTCAGGGCTAAGAGCGCAG | GCCCTCCAAGTTCCAAGACACATCC |
| TNFu1c1_F1 | TCCGCTGGTTGAATGATTCTTTCCC | GAGCACCTTCCATGTGCCAGACATC |
| TNFu4d2_F1 | AACCTGGGATTCAGGAATGTGTGGC | CACCCACAAGAAGAGGCAGATGCAG |
| TNIPc11_F | GAAGTGATGGCCAGGGGAGAACGTA | GGCAAAACTGAACTGGAGAGGCTGG |
| TNIPc12_F | GGAAGGAGTTCCCTGAGGATGACCC | AGGTTCCATGTGATTCCAAGCCGTC |
| TNIPc13_F | GAGGTGTAGCTGTCCCACTCCCCAT | CAGTTGCCCTTCCTGGGTCCAGTC |
| TNIPc14_F | TAGCAGAGAAGCAGCGAGGTTCACG | CTGCTACCTCCCTCAAGCCTCCTCA |
| TNIPc15_F | TGGTACCCAGCCTGCTAGCCTTCAG | ACCCAAACTCTGATACCCATGGCCT |
| TNIPc16_F | TCACTTGTCCCATTTTCCCCAGTCC | GACATCACCCCCAAACTCAGGCAAT |
| TNIPc17u3_F | TCACTGTTCCTGCACTGCATTCCATA | AGGGGGTTGACAGGGGAACATTAGC |
| TNIPc3_F | GAGCAGCAGCCAACTTGGGATTTTC | GCAACCTCCACCCATGACTCCAAAT |
| TNIPc4_F | ACAGTGCTGCTGGTTCAAGGTCAGC | TGAACCTCCTTCCTGTGTGCTCCAA |
| TNIPc5_F | AGTCCTGGGTTCTGCCCTGTGAGTC | TGAGAAATAAAGGCAGGGCTGCTGG |
| TNIPc6_F | CCCATGGCTGATACTTGGTCAGGCT | CAGTGGGGGAGCTCTCATGTTCTCA |
| TNIPc7_F | TGCGATAGTGAGCATTAAGGAGCCC | GAAGCCACAGTGACTACCAGCAGCC |
| TNIPc8_F | TCAGGGAGGGGGAATCATCATCTTG | CTCTCAAGGGTCCCTCCACTCCTCA |
| TNIPc9c10_F | GGTGGATGGCTTTGAGCAACATTGA | TCCGCTAATGTGGAGGGGAACTGAC |
| TNIPu1_F | TGTTCGCGGTAGAGTCTTTATGGGTC | ACAGGATAGACACCCAAAGGGCCG |
| TNIPu2c1c2_F | GCCTGCTGATATGCTGCTGTTGTCC | TGAAGGATTTGGGCAGTTTGGGGTA |
| VCAM1c2_F1 | CGGTGCTTTTTGGCTACCTTGCAGT | AAGTTGAGCGGTTGAGCACCAAGG |
| VCAM1c3_F1 | GCCCATCCATGTATTTGTTTGGCCT | CCACAAAGACTGGAACACATCGCAC |
| VCAM1c4_F1 | CCTCTGTTGCACTGGGATGATGCTT | GCTCAAATCCCACTGACAGCACCTT |
| VCAM1c5_F1 | TCATCTTGTTTTCCACCTGTGTCCCA | GGCATAAACCAAGGATTGTGGGTGC |
| VCAM1c6_F1 | CCATGGCACACAGCAAGATTGCATA | GCCTGGCAAACACGATAAAGAGACAGG |
| VCAM1c7_F1 | GGTCAAGCTAACAAGCGTCTCTGCC | TGACACAGGTCCTGAAGGCTTGCAT |
| VCAM1c8_F1 | CCAGGGAAGCTATTGTTGATGTCGC | CCACAGCTCCATTTTGCCAACTCAT |
| VCAM1c9u9_F1 | CTGCTTTGATTCCCTTCTCTTTGGAG | GAGCTACAATGTGACTAAAGGAGGCA |
| VCAM1u1c1_F1 | TCCGCCTCTGCAACAAGACCCTTTA | ACTGACTGAAGCCAAAACCAGGGCT |
| ZPBP2c3_F1 | CGTGTCTTCAGCACAAAATGGTAGGA | CAGCTCTGAGAAGCAGGGCACAAAA |
| ZPBP2c4_F1 | TGTGCCCTGCTTCTCAGAGCTGTAT | TCAGCACAACCCAGTGAAAGGATCA |
| ZPBP2c5_F1 | GCCAACCCACAGAAGCCATTTCTCT | TCACTGCATCTGGGGGCTAATGTAA |
| ZPBP2c6_F1 | GCACATGGAATTCAGCACTTGGACC | TTGGGGAACAAAGACCAGGGATCTA |
| ZPBP2c7_F1 | TCTTGCTTTCCATTTTGCTTGGCTC | CCACTGCAGAGGAAAACACAGGGAG |
| ZPBP2cu8d1_F1 | GGGCACTGCAGAAGATACTGTAAAATGCTG | GGGCGATGGAGCAAGACTCTGTTT |
| ZPBP2u1c2_F1 | GCGACACCGTGGCTTCTTTTCCTTT | TCCCAGCCCTTTGTGAGCTGTAAGG |
| ZPBP2u8d2_F1 | TGTGGAAGTCGGGGACATAAGATGA | TGGGCGATGGAGCAAGACTCTGTTT |
